# Metabolic phenotype dimensions and longitudinal renal function in type 2 diabetes: a principal component analysis-based approach with UACR integration

**DOI:** 10.3389/fendo.2026.1929060

**Published:** 2026-08-31

**Authors:** Caixia Mei, Yang Li, Hailin Ma, Xueling Li, Sheng Jiang

**Affiliations:** 1 Department of Endocrinology, The First Affiliated Hospital of Xinjiang Medical University, Urumqi, China; 2 Department of Endocrinology, Tongchuan District People’s Hospital of Dazhou, Dazhou, China

**Keywords:** cardiometabolic comorbidities, estimated glomerular filtration rate (eGFR), KDIGO risk stratification, metabolic phenotype, precision medicine, principal component analysis (PCA), renal function decline, type 2 diabetes (T2D)

## Abstract

**Background:**

Type 2 diabetes (T2D) exhibits substantial phenotypic heterogeneity, yet the relationship between multidimensional metabolic phenotypes and longitudinal renal function decline remains poorly characterized, particularly regarding albuminuria status.

**Objective:**

To identify metabolic phenotype dimensions using principal component analysis (PCA) in a Chinese T2D cohort and evaluate their associations with estimated glomerular filtration rate (eGFR) trajectories, rapid eGFR decline, cardiometabolic comorbidities, and KDIGO 2024 risk categories incorporating urinary albumin-to-creatinine ratio (UACR).

**Methods:**

In this retrospective cohort study of 1,427 patients from Dazhou Tongchuan District People’s Hospital (January 2024–July 2026), baseline metabolic variables were subjected to PCA with Varimax rotation (N = 621 with ≥6 variables). Multiple imputation by chained equations (MICE, 20 imputations) addressed missing data. UACR was calculated from simultaneously measured urine creatinine and microalbumin (374 observations). Longitudinal eGFR trajectories were analyzed using linear mixed models (LMM; N = 162, 415 observations), with UACR-adjusted sensitivity analysis (N = 103). Rapid eGFR decline (annual slope *<* −3 mL/min/1.73 m^2^/year) was predicted using logistic regression with bootstrap internal validation (1,000 resamples). Temporal validation was performed by comparing PCA structures derived from pre- versus post-intervention cohorts. The 3-component solution was compared against a 4-component alternative using predictive performance and loading stability. Subgroup analyses assessed the consistency of the months × PC1 interaction across age, sex, baseline eGFR, glycemic control, and sodium-glucose cotransporter-2 inhibitor (SGLT2i) use strata.

**Results:**

Three principal components explained 53.1% of total variance: PC1 (Blood Pressure Axis, 20.2%), PC2 (Metabolic Syndrome Axis, HDL−/TG+, 17.3%), and PC3 (Renal Function Axis, eGFR+/UA−, 15.6%). Parallel analysis suggested 4 components; the 4-component model improved predictive discrimination (AUC: 0.633 vs. 0.699; LRT *P* = 0.001), but PC4 was dominated by HbA1c and LDL-C without distinct physiological interpretation, and the 3component solution was retained on grounds of parsimony and interpretability. Temporal validation demonstrated excellent stability of PCA loading structures (Pre vs. Post: PC1 *r* = 0.961, PC2 *r* = 0.928, PC3 *r* = −0.976). Hopkins statistic (0.994) confirmed continuous data structure; MICE intraclass correlation coefficients ranged from 0.617 to 0.719. Compared with excluded patients, included patients had higher HbA1c (9.1% vs. 8.3%, *P <* 0.001), SBP (132 vs. 126 mmHg, *P <* 0.001), and longer diabetes duration (62 vs. 41 months, *P <* 0.001). In covariate-adjusted LMM, only PC1 was associated with differential eGFR trajectories (months × PC1: *β* = −0.135 per month, *P* = 0.028; annualized: −1.62 mL/min/1.73 m^2^/year per 1-SD PC1; approximately −3.55 mL/min/1.73 m^2^/year between extreme tertiles). This association was substantially attenuated after UACR adjustment (*P* = 0.792). Subgroup analyses showed a consistent direction of the months × PC1 interaction across all examined strata. PC1 was associated with macroalbuminuria (OR 1.76, *P* = 0.007) and PC3 was inversely associated with albuminuria (OR 0.65, *P* = 0.014). For rapid eGFR decline prediction (65/119, 54.6%), addition of PCA dimensions yielded a modest, non-significant improvement (AUC: 0.562 → 0.636; bootstrap-corrected: 0.674, 95% CI 0.588–0.762; LRT *P* = 0.315). In the UACR-available subcohort (N = 35, 18 events; exploratory only), AUC increased from 0.837 to 0.925; however, given the extremely small sample and quasi-complete separation, this value is almost certainly overestimated and should not be cited as evidence of predictive utility.

**Conclusions:**

In this single-center, short-term retrospective study, PCA-derived metabolic phenotype dimensions, particularly the blood pressure axis (PC1), were associated with differential eGFR trajectories and albuminuria status. The attenuation of the PC1–eGFR association after UACR adjustment generates the hypothesis that blood pressure-associated renal function decline may involve albuminuric pathways. Temporal validation confirmed PCA loading structure stability, though predictive model generalization was limited. These findings are hypothesis-generating given the observational design, limited UACR availability, short followup, and absence of independent external validation. Prospective, multi-center studies with comprehensive phenotyping are required before clinical translation can be considered.

## 1 Introduction

Type 2 diabetes (T2D) affects approximately 537 million adults worldwide and represents a leading cause of chronic kidney disease (CKD) and end-stage renal disease (1). Despite the recognition that T2D encompasses heterogeneous pathophysiological processes, clinical management has traditionally relied on unidimensional measures such as glycated hemoglobin (HbA1c) for risk stratification and treatment guidance (2).

Recent advances in diabetes phenotyping have demonstrated the value of data-driven approaches. Ahlqvist et al. (3) identified five novel subgroups of adult-onset diabetes using cluster analysis of six clinical variables, which showed differential risks of diabetic complications. Similarly, Wagner et al. (4) demonstrated pathophysiology-based subphenotyping of individuals at elevated risk for T2D. These studies underscore the importance of moving beyond glycemia-centric classification toward multidimensional phenotyping.

Principal component analysis (PCA) offers a complementary approach to cluster-based methods by identifying continuous latent dimensions that capture the covariance structure among metabolic variables (5). Unlike discrete clustering approaches (e.g., *k*-means, latent profile analysis [LPA]), which partition patients into mutually exclusive subgroups, PCA preserves the continuous nature of metabolic derangements. This distinction is clinically important for three reasons. First, T2D metabolic abnormalities exist along continuous spectra rather than as discrete categories—a premise confirmed in our dataset by a Hopkins statistic of 0.994, which strongly favors a continuous over a clustered data structure. Second, PCA generates interpretable axes (e.g., a “blood pressure axis” dominated by systolic blood pressure [SBP] and diastolic blood pressure [DBP]) that correspond to distinct pathophysiological domains, facilitating mechanistic interpretation and clinical communication. Third, continuous PCA scores enable dose-response analyses and threshold-free risk assessment, avoiding the information loss inherent in arbitrary categorization. While LPA identified four clusters in this dataset (**Supplementary Figure 1**), the Hopkins statistic strongly favored a dimensional structure, supporting PCA as the more appropriate analytical framework. This approach has been successfully applied in metabolomics (6) and cardiovascular research (7), but its application to routine clinical variables for renal function prediction in T2D remains underexplored.

The relationship between metabolic phenotypes and renal function decline is of particular clinical importance. CKD affects approximately 20–40% of patients with T2D (8), and eGFR decline is a strong predictor of cardiovascular events and mortality (9). The Kidney Disease: Improving Global Outcomes (KDIGO) 2024 guidelines emphasize the combined use of eGFR and urinary albumin-to-creatinine ratio (UACR) for CKD risk stratification (8). While traditional risk factors such as hypertension, glycemic control, and albuminuria are well-established predictors of CKD progression (10), the extent to which multidimensional metabolic phenotypes—capturing the interplay between blood pressure, dyslipidemia, obesity, and renal function— are associated with differential eGFR trajectories, and whether these associations are independent of albuminuria, remains poorly characterized.

Furthermore, the emergence of sodium-glucose cotransporter-2 inhibitors (SGLT2i) as renoprotective agents (11, 12) raises the question of whether the renal benefit of these medications varies across metabolic phenotype dimensions.

In the present study, we applied PCA with Varimax rotation to routine clinical and laboratory variables from a Chinese T2D cohort to: (1) identify distinct metabolic phenotype dimensions; (2) evaluate their cross-sectional associations with cardiometabolic comorbidities including UACR-defined outcomes; (3) assess their longitudinal associations with eGFR trajectories, with and without UACR adjustment; (4) examine potential interactions between phenotype dimensions and SGLT2i use and uric acid status; and (5) evaluate the incremental predictive value of PCA dimensions for rapid eGFR decline. The PCA approach was rigorously validated through parallel analysis (13), Promax oblique rotation sensitivity, comparison with a 4-component alternative, the Hopkins statistic (14), LPA, MICEderived intraclass correlation coefficients (ICC) (15), complete-case sensitivity analyses, and temporal validation across pre- and post-intervention cohorts.

## 2 Methods

### 2.1 Study design and population

This was a retrospective cohort study using electronic health records from Dazhou Tongchuan District People’s Hospital (Sichuan Province, China). The study period spanned from January 1, 2024, to July 1, 2026. The study was approved by the institutional ethics committee, and the requirement for informed consent was waived for this retrospective analysis of de-identified data.

A total of 1,427 patients with T2D contributed 2,088 observation records. Inclusion required at least one valid HbA1c measurement. For the PCA analysis, patients were required to have at least 6 of 9 key metabolic variables available at baseline (N = 621). For longitudinal eGFR analysis, patients from the PCA cohort with at least 2 eGFR measurements were included (N = 162, 415 observations). A comparison of included versus excluded participants was performed to assess potential selection bias (**Supplementary Table 1**).

### 2.2 Variables and measurements

The following baseline variables were extracted: HbA1c (%), body mass index (BMI, kg/m^2^), SBP.

(mmHg), DBP (mmHg), low-density lipoprotein cholesterol (LDL-C, mmol/L), high-density lipoprotein cholesterol (HDL-C, mmol/L), triglycerides (TG, mmol/L), eGFR (mL/min/1.73 m^2^), and uric acid (UA, *µ*mol/L). eGFR was calculated using the 2021 CKD-EPI creatinine equation refit without the race coefficient (16).

UACR (mg/g) was calculated from simultaneously measured urine creatinine and microalbumin as **Equation 1**:

(1) $$\text{UACR}=\frac{\text{urine microalbumin }(\text{mg}/\text{L})}{\text{urine creatinine }(\text{μmol}/\text{L})}\times 8840$$

KDIGO 2024 risk categories were defined by cross-tabulating eGFR and UACR (8). Medication data were extracted through text parsing of clinical notes and were limited to drug class (binary indicator) without information on dose, duration, or adherence. Renin-angiotensin-aldosterone system (RAAS) inhibitor and statin use were not specifically captured. Comorbidities were identified through diagnostic codes and clinical thresholds.

### 2.3 Principal component analysis

All nine metabolic variables were winsorized at the 1st and 99th percentiles to reduce the influence of extreme values (**Supplementary Table 2**) and standardized to *z*-scores. MICE (20 imputations, 50 iterations) was performed to address missing data (17). PCA with Varimax (orthogonal) rotation was applied to the imputed data matrix. The number of retained components was determined by eigenvalues *>* 1 (Kaiser criterion), scree plot inspection, parallel analysis with 100 iterations (13), and interpretability. Component scores were computed for each patient using the regression method.

As sensitivity analyses, Promax (oblique) rotation was applied to assess whether allowing correlated factors substantially altered the loading structure. A 4-component solution was also examined per the parallel analysis result (**Supplementary Table 3**). The 3-component and 4-component models were compared on variance explained, predictive performance (AUC for rapid eGFR decline), loading stability, cross-loading patterns (number of variables with absolute loadings *>* 0.3 on multiple PCs), and biological interpretability (**Supplementary Table 4**). Robustness was further evaluated through complete-case sensitivity analysis (N = 265 with all 9 variables) and MICE-derived ICC (two-way agreement model) across 20 imputations (15).

### 2.4 Data structure validation

The Hopkins statistic was calculated to assess clustering tendency, with values *>* 0.5 indicating a continuous data structure suitable for PCA (14). LPA with 1–6 Gaussian mixture models was performed as a complementary approach, with the optimal number of clusters selected by the Bayesian Information Criterion (BIC).

### 2.5 Temporal validation

To assess the stability of PCA-derived dimensions across time periods, the cohort was divided into pre-intervention (baseline visit before June 1, 2025) and post-intervention (baseline visit on or after June 1, 2025) subsets. PCA was performed independently on each subset, and loading correlations were computed to quantify structural stability. A logistic regression model for rapid eGFR decline trained on the preintervention cohort was externally applied to the post-intervention cohort to evaluate predictive model generalizability.

### 2.6 Cross-sectional comorbidity analysis

Eight binary comorbidity outcomes were defined: hypertension (documented diagnosis or SBP ≥ 140 mmHg or DBP ≥ 90 mmHg), CKD (eGFR *<* 60 mL/min/1.73 m^2^), hyperuricemia (UA *>* 420 *µ*mol/L for males, *>* 360 *µ*mol/L for females), dyslipidemia (LDL-C *>* 2.6 mmol/L or TG *>* 1.7 mmol/L), obesity (BMI ≥ 28 kg/m^2^), severe hyperglycemia (HbA1c *>* 9%), albuminuria (UACR ≥ 30 mg/g), and macroalbuminuria (UACR ≥ 300 mg/g). Multivariable logistic regression models were fit for each comorbidity with PC1, PC2, and PC3 as predictors, adjusted for age and sex. Odds ratios (OR) per 1 SD increment in PC score were reported with 95% confidence intervals (CI).

### 2.7 Longitudinal eGFR analysis

Linear mixed models (LMM) were used to assess the association between PCA dimensions and eGFR trajectories. The primary model included fixed effects for months (continuous time from first visit), PC1, PC2, PC3, age, sex, HbA1c, SGLT2i use, and all two-way interactions between months and each PC. Random intercepts were specified at the patient level (random slopes resulted in a singular fit and were simplified to intercept-only). The Benjamini-Hochberg false discovery rate (FDR) correction was applied to interaction terms.

Annualized effect sizes were calculated by multiplying the monthly interaction coefficient by 12. The expected annual eGFR difference between the highest and lowest PC1 tertiles was estimated by multiplying the annualized coefficient by the range of mean PC1 scores across tertiles.

Secondary models explored three-way interactions: (1) months × PC1 × uric acid status and (2) months × PC2 × SGLT2i use. Stratified LMM quantified SGLT2i effects within each PC2 tertile (**Supplementary Table 5**). Subgroup analyses of the months × PC1 interaction were performed across age (median-split), sex, baseline eGFR (≥ 60 vs. *<* 60 mL/min/1.73 m^2^), glycemic control (HbA1c ≥ 8% vs. *<* 8%), and SGLT2i use (**Supplementary Table 6**).

A UACR-adjusted sensitivity model additionally included log UACR and months × log UACR as fixed effects, using all available observations with non-missing UACR. Given the limited UACR data (17.9% of observations), this analysis should be considered exploratory.

### 2.8 Rapid eGFR decline prediction

The annual eGFR slope was calculated using ordinary least squares regression for patients with ≥3 eGFR measurements, and the difference between the last and first eGFR divided by time interval for patients with exactly 2 measurements. Slopes were winsorized at the 2.5th and 97.5th percentiles. Rapid decline was defined as an annual slope *<* −3 mL/min/1.73 m^2^/year (18). Two logistic regression models were compared: a base model (age + sex + baseline eGFR) and an augmented model (base + PC1 + PC2 + PC3). Model discrimination was assessed using the area under the receiver operating characteristic curve (AUC), with internal validation by bootstrap resampling (1,000 iterations). Continuous and categorical net reclassification improvement (NRI; categories: *<*30%, 30–60%, *>*60% predicted risk), integrated discrimination improvement (IDI), the likelihood ratio test (LRT), and the HosmerLemeshow (HL) goodness-of-fit test were calculated (**Supplementary Table 7**). Calibration was assessed visually (**Supplementary Figure 2**). Decision curve analysis (DCA) evaluated net benefit across threshold probabilities of 5–50%. A sensitivity analysis excluded patients with less than 6 months of follow-up.

In the UACR-available subcohort, prediction models additionally included log UACR as a predictor (**Supplementary Table 8**). These analyses are exploratory and should not be used for clinical inference.

### 2.9 Patient engagement analysis

As an exploratory bridge analysis, PCA dimension scores were compared across three WeChat-based follow-up groups (G1: consistent, G2: new, G3: limited) using ANOVA and ANCOVA adjusting for age, sex, baseline HbA1c, and baseline SBP. Given the cross-sectional nature of this analysis, bidirectional confounding between metabolic burden and healthcare engagement cannot be excluded.

### 2.10 Software and reproducibility

All statistical analyses were performed using R version 4.2–4.4 with packages including psych, mice, lme4/lmerTest, pROC, boot, mclust, factoextra, nFactors, GPArotation, and irr. Statistical significance was set at a two-sided *P <* 0.05 unless otherwise specified. Complete PCA loading matrices and model specifications are provided in the Supplementary Material to facilitate independent replication.

## 3 Results

### 3.1 Patient characteristics and PCA structure

Among 1,427 patients, 621 (43.5%) had at least 6 of 9 key metabolic variables for PCA (265 complete cases). For longitudinal eGFR, 162 patients contributed 415 observations.

Comparison of included versus excluded participants (**Supplementary Table 1**) revealed that PCAeligible patients had higher HbA1c (9.1% vs. 8.3%, *P <* 0.001), higher SBP (132 vs. 126 mmHg, *P <* 0.001), higher DBP (85 vs. 80 mmHg, *P <* 0.001), and longer diabetes duration (62 vs. 41 months, *P <* 0.001), suggesting that the PCA cohort was biased toward patients with more complete clinical data and possibly greater metabolic disease severity. Age, BMI, eGFR, and UA did not differ significantly between groups.

**Figure 1:**
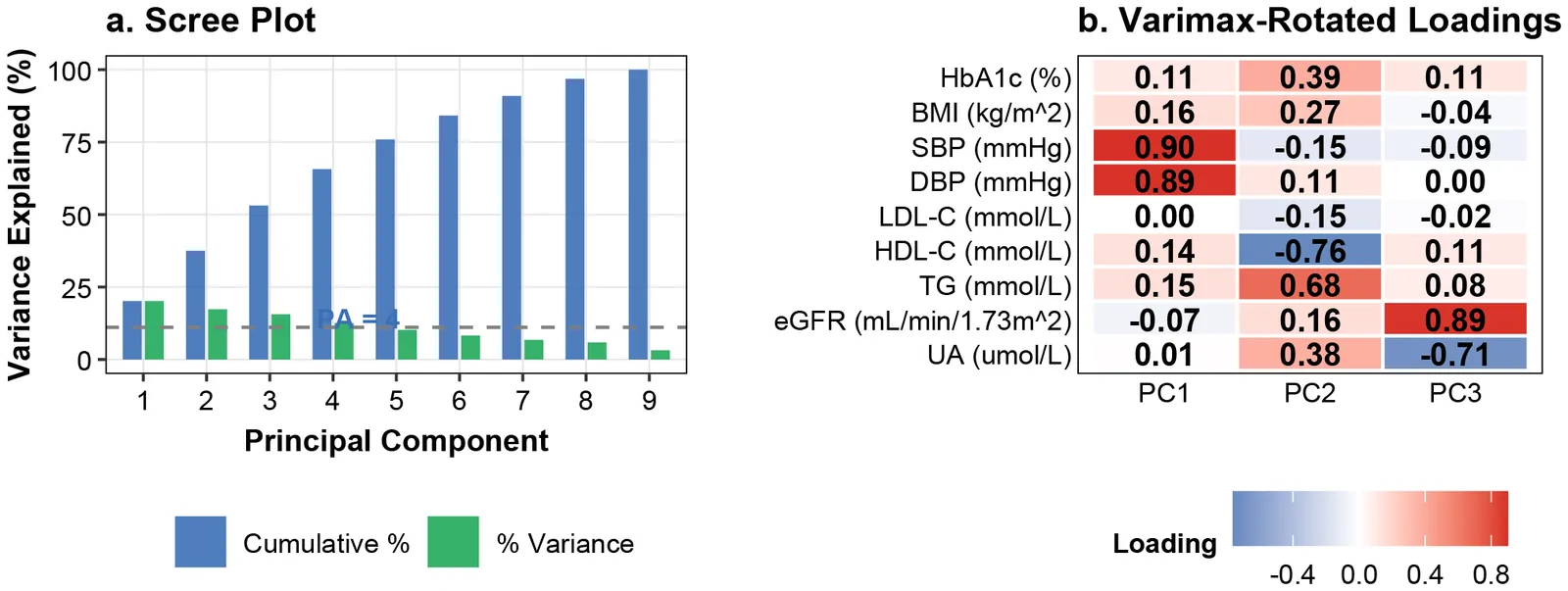
PCA structure of metabolic phenotype dimensions. PCA Structure of Metabolic Phenotype Dimensions. **(a)** Scree plot showing variance explained by each principal component. Dashed horizontal line indicates the Kaiser criterion (eigenvalue = 1). The annotation “PA = 4” indicates that parallel analysis (100 iterations) suggested 4 components as the upper bound. **(b)** Heatmap of Varimax-rotated factor loadings. PC1 (Blood Pressure Axis) is dominated by SBP and DBP; PC2 (Metabolic Syndrome Axis) is dominated by low HDL-C and high TG; PC3 (Renal Function Axis) is dominated by high eGFR and low UA. Loadings are displayed with color intensity and numerical values. N = 621. Abbreviations: SBP, systolic blood pressure; DBP, diastolic blood pressure; HDL-C, high-density lipoprotein cholesterol; TG, triglycerides; eGFR, estimated glomerular filtration rate; UA, uric acid; BMI, body mass index; LDL-C, low-density lipoprotein cholesterol.

Hopkins statistic of 0.994 confirmed a continuous data structure, supporting the appropriateness of PCA over discrete clustering. PCA with Varimax rotation identified three components with eigenvalues *>* 1, explaining 53.1% of total variance: PC1 (Blood Pressure Axis, 20.2%), PC2 (Metabolic Syndrome Axis, 17.3%), and PC3 (Renal Function Axis, 15.6%). Parallel analysis suggested 4 components as the upper bound (**Figure 1a**).

**Figure 2:**
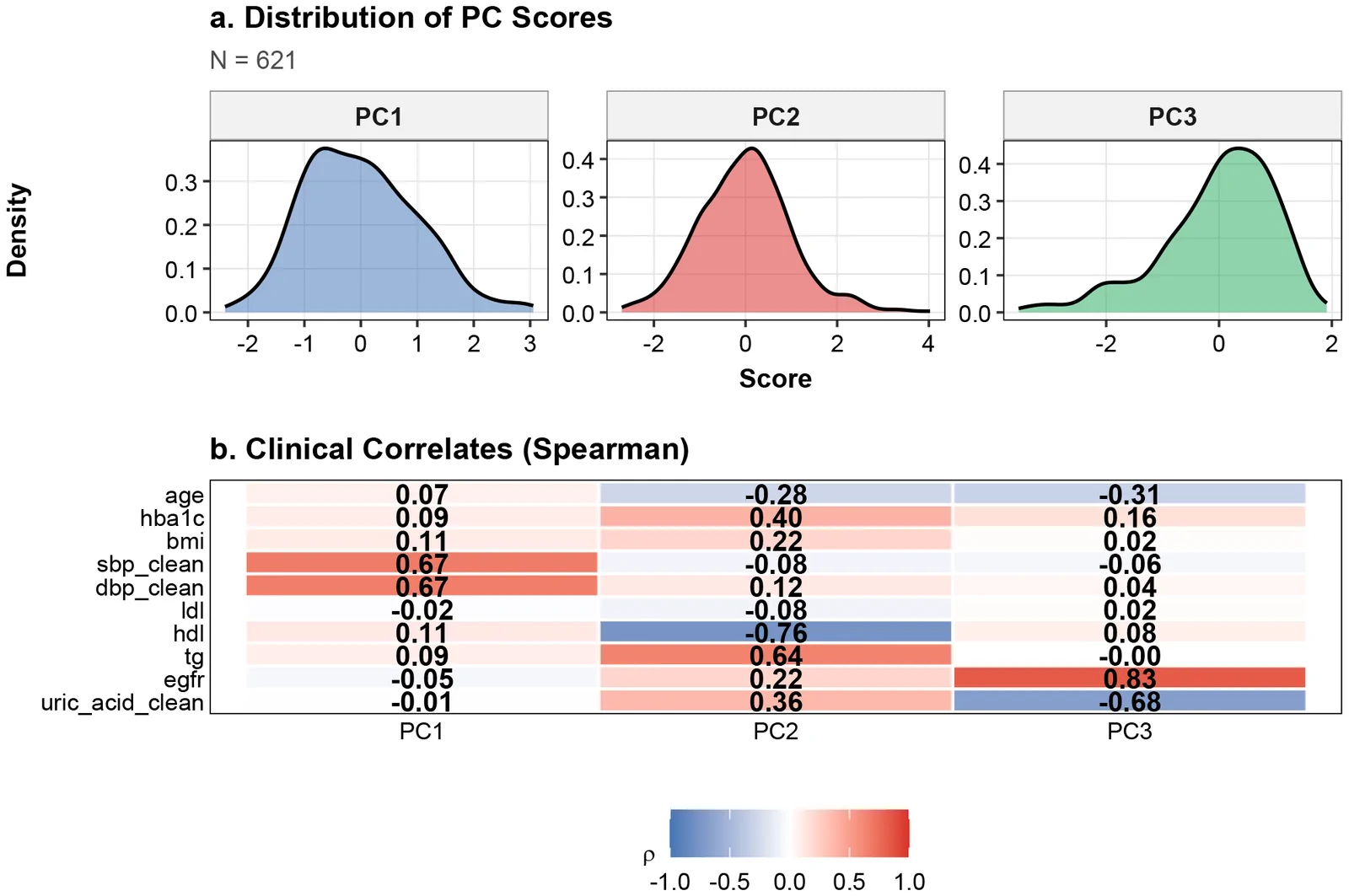
PC score distributions and clinical correlates. PC Score Distributions and Clinical Correlates (N = 621). **(a)** Density distributions of PC1, PC2, and PC3 scores, demonstrating approximately normal distributions consistent with PCA assumptions derived from *z*-score-standardized input variables. **(b)** Spearman correlation heatmap between clinical variables and PC scores. The pattern of correlations confirms the clinical interpretation: PC1 correlates strongly with SBP and DBP; PC2 correlates negatively with HDL-C and positively with TG, HbA1c, and UA; PC3 correlates positively with eGFR and negatively with UA. Age was included as a clinical reference variable and was not part of the PCA input matrix.

Loading structure (**Figure 1b**; **Table 1**): PC1 dominated by SBP (+0.900) and DBP (+0.894); PC2 by low HDL-C (−0.763) and high TG (+0.678); PC3 by high eGFR (+0.888) and low UA (−0.708). The 4-component solution (**Supplementary Table 3**) showed PC4 explaining 12.6% of variance, dominated by HbA1c (+0.768) and LDL-C (+0.652). Although the 4-component model provided a statistically significant improvement in rapid eGFR decline prediction (AUC: 0.633 vs. 0.699; LRT *P* = 0.001), PC4 was dominated by variables already represented in PC2, introduced cross-loadings (HbA1c and BMI loading *>* 0.3 on multiple PCs), and lacked a distinct physiological interpretation. A complete comparison of the two solutions is provided in **Supplementary Table 4**. On grounds of parsimony and interpretability, the 3-component solution was retained. PC score distributions approximated normal distributions, and their correlations with clinical variables confirmed the clinical interpretation of each dimension (**Figure 2**): PC1 correlated strongly with SBP and DBP; PC2 correlated negatively with HDL-C and positively with TG, HbA1c, and UA; and PC3 correlated positively with eGFR and negatively with UA.

**Table 1:** Varimax-rotated PCA loadings.

Variable	PC1	PC2	PC3
HbA1c (%)	0.107	0.387	0.114
BMI (kg/m^2^)	0.157	0.269	–0.042
SBP (mmHg)	0.900	–0.145	–0.088
DBP (mmHg)	0.894	0.110	0.000
LDL-C (mmol/L)	0.003	–0.146	–0.022
HDL-C (mmol/L)	0.140	–0.763	0.105
TG (mmol/L)	0.147	0.678	0.084
eGFR (mL/min/1.73 m^2^)	–0.074	0.159	0.888
UA ( μmol/L)	0.007	0.376	–0.708

N = 621. PC1 = 20.2%, PC2 = 17.3%, PC3 = 15.6% variance explained. Cumulative = 53.1%. Parallel Analysis suggested 4 components. Promax max |*r*| = 0.059. Varimax-Promax loading correlations: PC1 = 0.998, PC2 = −0.162, PC3 = 0.002. MICE ICC: PC1 = 0.617, PC2 = 0.673, PC3 = 0.719. Complete-case loading correlations: PC1 = 0.988, PC2 = 0.964, PC3 = 0.872.

Sensitivity analyses: MICE-derived ICC values were 0.617 (PC1), 0.673 (PC2), and 0.719 (PC3). Complete-case loading correlations with the primary PCA were *r* = 0.988, 0.964, and 0.872 for PC1, PC2, and PC3, respectively. Promax oblique rotation yielded near-orthogonal dimensions (inter-factor correlations: max |*r*| = 0.059). Varimax-Promax loading correlations were 0.998 (PC1), −0.162 (PC2), and 0.002 (PC3), confirming that PC1 was rotation-invariant while PC2 and PC3 showed some sensitivity to the choice of rotation.

### 3.2 Temporal validation of PCA structure

Temporal validation comparing pre-intervention (N = 356) and post-intervention (N = 265) cohorts demonstrated excellent stability of PCA loading structures: PC1 *r* = 0.961, PC2 *r* = 0.928, and PC3 *r* = −0.976, where the sign reversal for PC3 is attributable to the known sign ambiguity of PCA. For rapid eGFR decline prediction, the model trained on the pre-intervention cohort (N = 76, 42 events) yielded AUC of 0.551 (base) and 0.635 (+PC). When applied to the post-intervention cohort (N = 42, 22 events), AUC was 0.575 (base) and 0.543 (+PC), indicating that the incremental predictive value of PCA dimensions did not generalize across time periods (generalization gap: −0.092 for +PC model; **Supplementary Figure 3**).

### 3.3 UACR characteristics

Baseline UACR was available in 184 patients (29.6% of PCA cohort). Median UACR was 22 mg/g (IQR 8–102). KDIGO A1, A2, and A3 categories comprised 57.6%, 27.7%, and 14.7% of those with available data, respectively (**Supplementary Table 6**). A total of 190 patients had complete eGFR and UACR data for KDIGO risk stratification (**Supplementary Figure 4**).

### 3.4 Cross-sectional comorbidity associations

**Figure 3:**
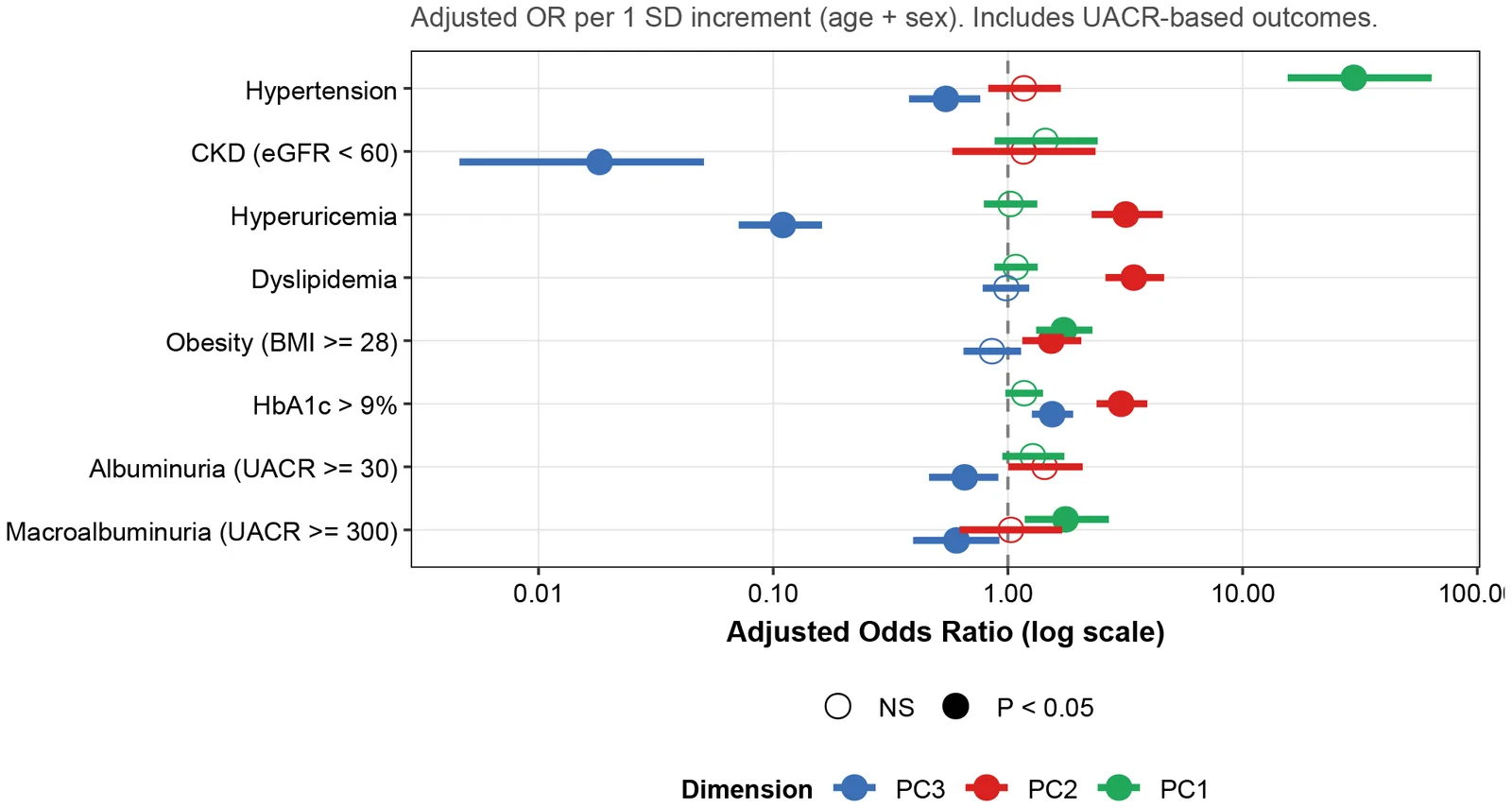
PC scores and cardiometabolic comorbidities. PC Scores and Cardiometabolic Comorbidities Including UACR Outcomes. Forest plot displaying adjusted OR (95% CI) per 1 SD increment in PC score, derived from multivariable logistic regression models adjusted for age and sex. Filled circles: *P <* 0.05; open circles: nonsignificant. PC1 is associated with hypertension (OR ≈ 30), obesity, and macroalbuminuria, confirming its interpretation as a blood pressure/hemodynamic axis. PC2 is associated with dyslipidemia, hyperuricemia, and poor glycemic control (HbA1c *>* 9%), reflecting the metabolic syndrome dimension. PC3 is inversely associated with CKD, hyperuricemia, and albuminuria, consistent with its interpretation as a renal function axis. The inclusion of UACR-based outcomes (albuminuria and macroalbuminuria) extends the clinical characterization of each dimension into the domain of diabetic kidney disease. Abbreviations: OR, odds ratio; CI, confidence interval; CKD, chronic kidney disease; BMI, body mass index; UACR, urinary albumin-to-creatinine ratio.

Associations between PCA dimensions and comorbidities are shown in **Figure 3** and **Table 2**. PC1 was associated with hypertension (OR 29.76 per 1 SD, 95% CI 15.56–63.88, *P <* 0.001), obesity (OR 1.73, *P <* 0.001), and macroalbuminuria (OR 1.76, 95% CI 1.18–2.69, *P* = 0.007). The association with moderate albuminuria was non-significant (OR 1.28, *P* = 0.114). PC2 was associated with dyslipidemia (OR 3.44), hyperuricemia (OR 3.18), and HbA1c *>* 9% (OR 3.04). PC3 was inversely associated with CKD (OR 0.02), hyperuricemia (OR 0.11), albuminuria (OR 0.65, *P* = 0.014), and macroalbuminuria (OR 0.60, *P* = 0.018).

**Table 2:** Comorbidity odds ratios per PC dimension.

Condition	PC	OR (95% CI)	*P*
Hypertension	PC1	29.76 (15.56–63.88)	<0.001
Hypertension	PC2	1.17 (0.82–1.68)	0.385
Hypertension	PC3	0.54 (0.38–0.76)	<0.001
CKD (eGFR < 60)	PC1	1.44 (0.88–2.41)	0.149
CKD (eGFR < 60)	PC2	1.17 (0.58–2.36)	0.664
CKD (eGFR < 60)	PC3	0.02 (0.00–0.05)	<0.001
Hyperuricemia	PC1	1.03 (0.79–1.33)	0.850
Hyperuricemia	PC2	3.18 (2.27–4.56)	<0.001
Hyperuricemia	PC3	0.11 (0.07–0.16)	<0.001
Dyslipidemia	PC1	1.08 (0.88–1.34)	0.474
Dyslipidemia	PC2	3.44 (2.60–4.63)	<0.001
Dyslipidemia	PC3	0.98 (0.78–1.23)	0.891
Obesity (BMI ≥ 28)	PC1	1.73 (1.32–2.29)	<0.001
Obesity (BMI ≥ 28)	PC2	1.53 (1.15–2.05)	0.004
Obesity (BMI ≥ 28)	PC3	0.85 (0.65–1.14)	0.273
HbA1c > 9%	PC1	1.17 (0.97–1.41)	0.093
HbA1c > 9%	PC2	3.04 (2.39–3.93)	<0.001
HbA1c > 9%	PC3	1.54 (1.26–1.90)	<0.001
Albuminuria (UACR ≥ 30)	PC1	1.28 (0.95–1.74)	0.114
Albuminuria (UACR ≥ 30)	PC2	1.43 (1.00–2.08)	0.052
Albuminuria (UACR ≥ 30)	PC3	0.65 (0.46–0.91)	0.014
Macroalbuminuria (UACR ≥ 300)	PC1	1.76 (1.18–2.69)	0.007
Macroalbuminuria (UACR ≥ 300)	PC2	1.03 (0.62–1.70)	0.911
Macroalbuminuria (UACR ≥ 300)	PC3	0.60 (0.39–0.92)	0.018

Adjusted for age and sex. OR per 1 SD increment in PC score. CI: 95% confidence interval. CKD defined as eGFR < 60 mL/min/1.73 m^2^; hyperuricemia as UA > 420 μmol/L (male) or > 360 μmol/L (female); dyslipidemia as LDL-C > 2.6 mmol/L or TG > 1.7 mmol/L; obesity as BMI ≥ 28 kg/m^2^; albuminuria as UACR ≥ 30 mg/g; macroalbuminuria as UACR ≥ 300 mg/g.

### 3.5 Medication use

**Figure 4:**
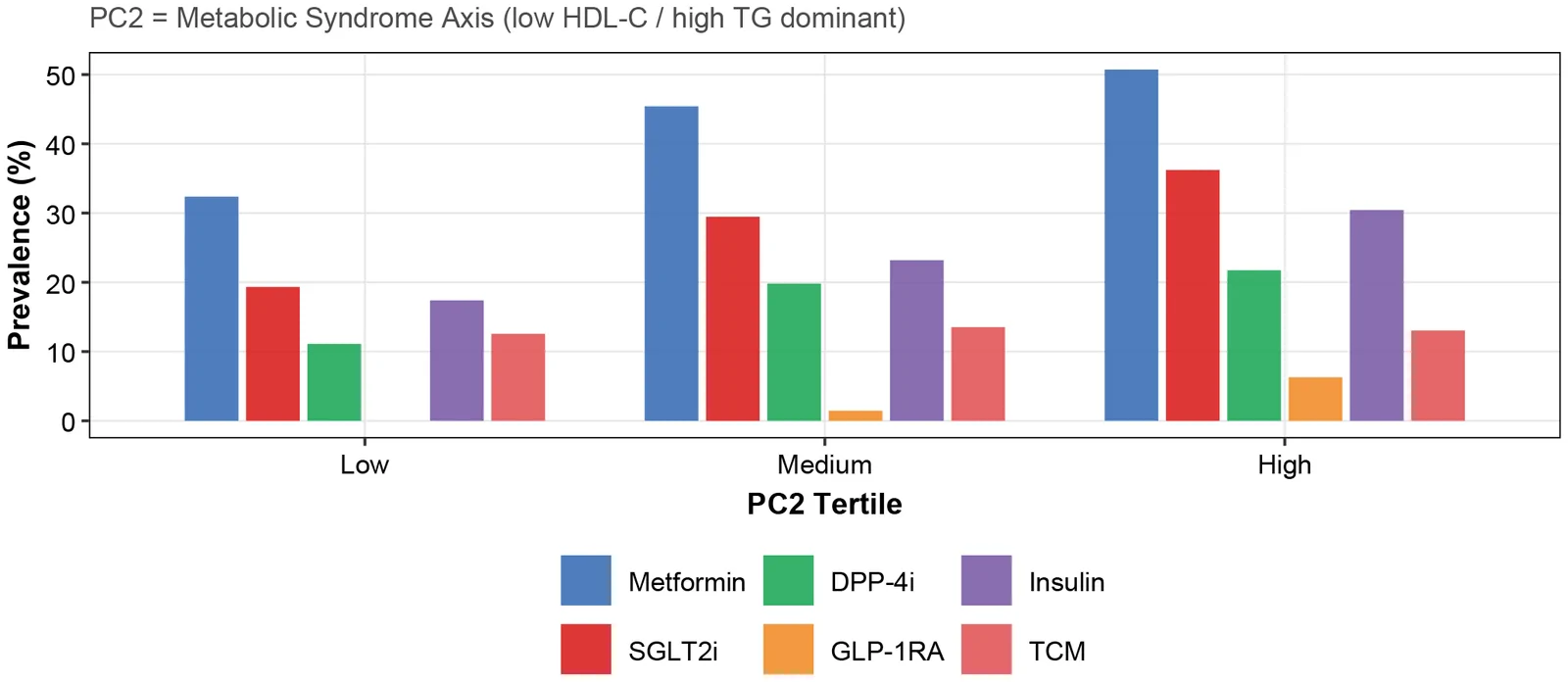
Medication use by PC2 tertile. Medication Use by PC2 Tertile. PC2 represents the Metabolic Syndrome Axis (low HDL-C, high TG). Higher PC2 tertiles were associated with numerically greater SGLT2i and GLP-1RA use, consistent with guideline recommendations prioritizing these agents in patients with metabolic comorbidities. Overall SGLT2i prescription rate was 52%. Medication data were derived from text parsing of clinical notes and reflect binary (ever-used) classification without information on dose, duration, or adherence. Abbreviations: SGLT2i, sodium-glucose cotransporter-2 inhibitor; DPP-4i, dipeptidyl peptidase-4 inhibitor; GLP-1RA, glucagon-like peptide-1 receptor agonist; TCM, traditional Chinese medicine.

Higher PC2 scores were associated with numerically greater SGLT2i and GLP-1 receptor agonist (GLP1RA) use (**Figure 4**). The overall SGLT2i prescription rate was 52%. Medication prevalence across PC1 tertiles showed that SGLT2i use increased from 26.6% (low PC1) to 32.9% (high PC1), and insulin use from 18.8% to 26.6%. Metformin, dipeptidyl peptidase-4 (DPP-4) inhibitor, GLP-1RA, and traditional Chinese medicine (TCM) use did not show clear trends across PC1 strata. The mean number of glucose-lowering medication classes was 1.18 (low PC1), 1.31 (medium PC1), and 1.35 (high PC1). Data on RAAS inhibitor and statin use were not available.

### 3.6 Longitudinal eGFR trajectories

**Figure 5:**
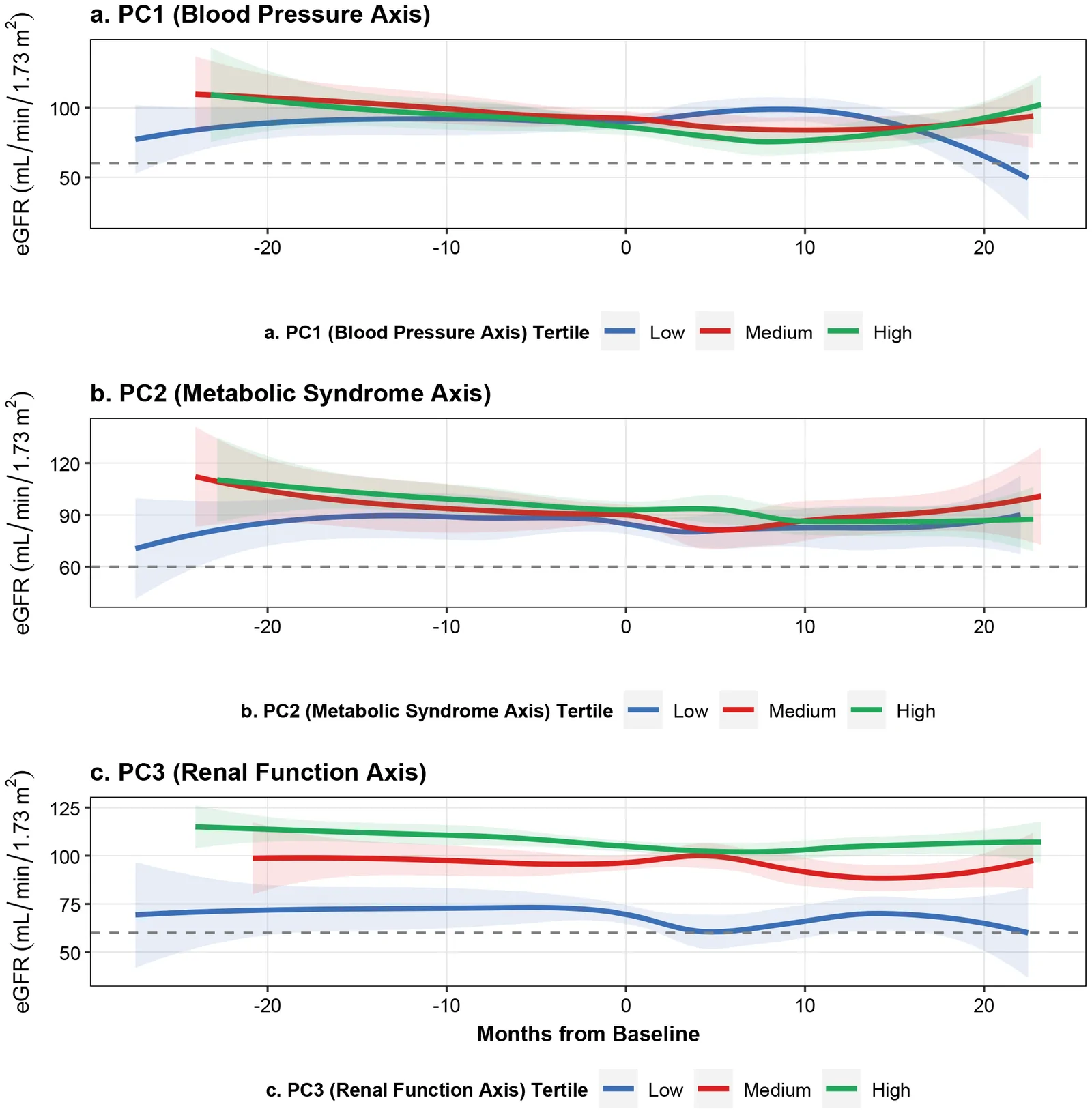
eGFR trajectories by PCA dimension tertile (N = 162). eGFR Trajectories by PCA Dimension Tertile (N = 162, 415 observations). Loesssmoothed trajectories with 95% confidence intervals (shaded). Dashed horizontal line: eGFR = 60 mL/min/1.73 m^2^ (threshold for CKD). **(a)** PC1 (Blood Pressure Axis): higher PC1 tertile was associated with steeper eGFR decline (months × PC1 interaction: *β* = −0.135 per month, *P* = 0.028; annualized: −1.62 mL/min/1.73 m^2^/year per 1-SD PC1). **(b)** PC2 (Metabolic Syndrome Axis): *P* = 0.909. **(c)** PC3 (Renal Function Axis): *P* = 0.758. The separation of trajectories by PC3 tertile reflects the crosssectional association between PC3 and baseline eGFR level rather than differential longitudinal decline. Abbreviations: eGFR, estimated glomerular filtration rate; CKD, chronic kidney disease; PC, principal component; SD, standard deviation.

In LMM analysis (**Table 3**), PC3 was the dominant cross-sectional predictor of eGFR level (*β* = +16.69, *P <* 0.001), confirming its interpretation as the Renal Function Axis. Only PC1 was associated with differential eGFR trajectories (months × PC1: *β* = −0.135 per month, *P* = 0.028; FDR-corrected *P* = 0.084; **Figures 5a–c**). This translates to an annualized decline of −1.62 mL/min/1.73 m^2^/year per 1-SD increment in PC1 score, or approximately −3.55 mL/min/1.73 m^2^/year between the highest and lowest PC1 tertiles. While this difference is statistically significant, its clinical significance at the individual patient level is uncertain given the short follow-up (∼13 months) and the wide within-person variability of eGFR measurements.

**Table 3:** Linear mixed model fixed effects for eGFR.

Term	Estimate	SE	*t*	*P*
(Intercept)	108.605	6.094	17.822	<0.001
months	–0.204	0.073	–2.784	0.006
PC1	–1.971	0.878	–2.245	0.026
PC2	1.274	0.876	1.454	0.148
PC3	16.689	1.007	16.567	<0.001
age	–0.416	0.098	–4.251	<0.001
sex (Male)	2.019	1.979	1.020	0.309
HbA1c	0.527	0.268	1.969	0.050
SGLT2i use	–1.541	1.344	–1.146	0.253
months × PC1	–0.135	0.061	–2.207	**0.028**
months × PC2	–0.006	0.056	–0.115	0.909
months × PC3	0.023	0.073	0.309	0.758

162 patients, 415 observations. Random intercept model (singular fit with random slopes corrected to intercept-only). Satterthwaite approximation for degrees of freedom. Annualized months × PC1 effect: −1.62 mL/min/1.73 m^2^/year per 1-SD PC1; approximately −3.55 mL/min/1.73 m^2^/year between highest and lowest PC1 tertiles. UACR-adjusted sensitivity (N = 103): months × PC1 P=0.792; months × log UACR P=0.626. Note: UACR data available in only 5.9% of baseline patients; UACR-adjusted results are exploratory. Bold: P<0.05.

**Figure 6:**
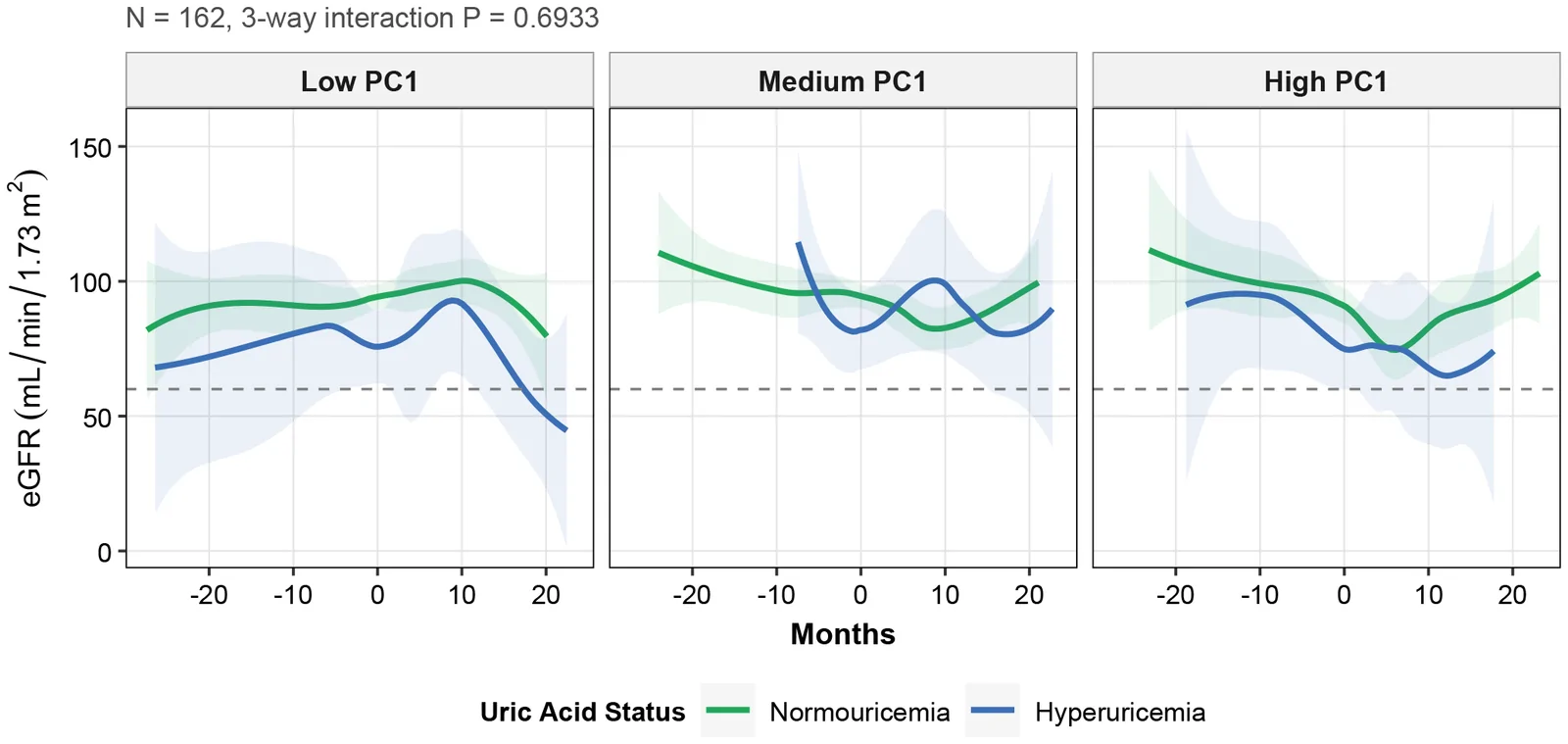
eGFR trajectories by PC1 tertile and uric acid status. eGFR Trajectories by PC1 Tertile and Uric Acid Status (N = 162). Three-way interaction (months × PC1 × UA): *P* = 0.693. Hyperuricemia was defined as UA *>* 420 *µ*mol/L for males or *>* 360 *µ*mol/L for females. The non-significant three-way interaction suggests that the association between PC1 and eGFR decline was not substantially modified by uric acid status over the observed follow-up period. Abbreviations: UA, uric acid; eGFR, estimated glomerular filtration rate; PC1, principal component 1 (Blood Pressure Axis).

**Figure 7:**
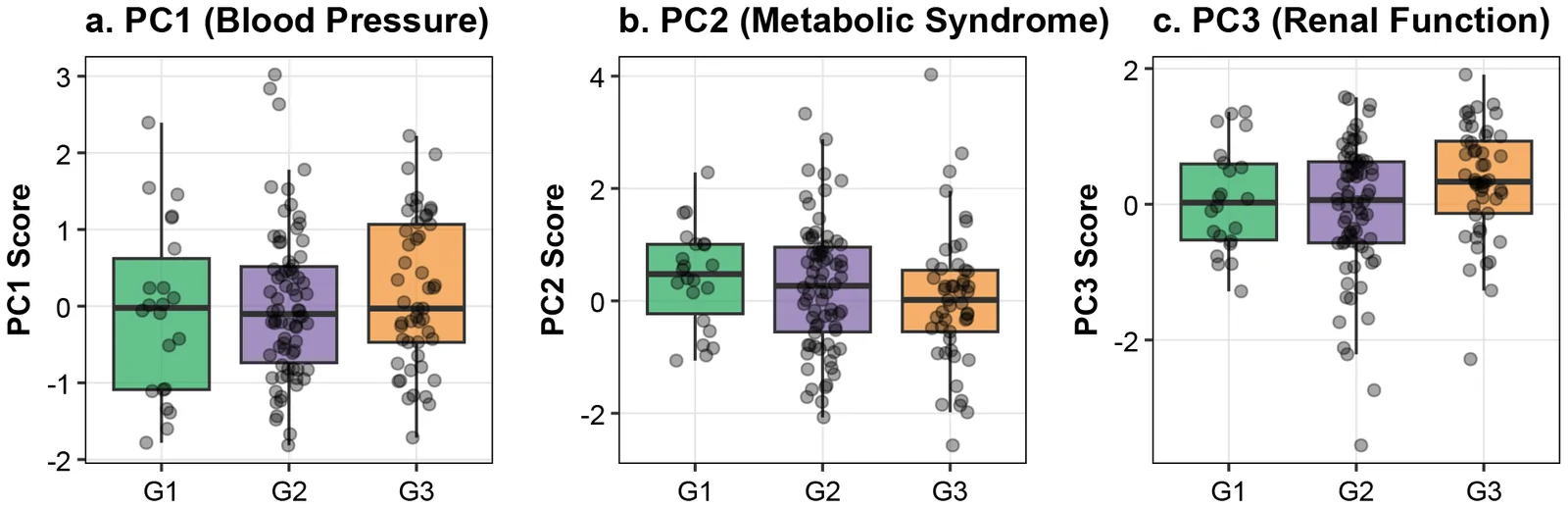
eGFR trajectories by PC2 tertile and SGLT2i use. eGFR Trajectories by PC2 Tertile and SGLT2i Use. Three-way interaction (months × PC2 × SGLT2i): *P* = 0.085 (trend-level). Stratified LMM results are presented in **Supplementary Table 3**. The pattern of eGFR trajectories suggests possible differential SGLT2i effects across metabolic phenotype strata, particularly a trend toward renoprotection in the medium PC2 tertile; however, all nominal *P*-values exceeded 0.05 and these analyses are underpowered. Abbreviations: SGLT2i, sodium-glucose cotransporter2 inhibitor; eGFR, estimated glomerular filtration rate; PC2, principal component 2 (Metabolic Syndrome Axis); LMM, linear mixed model.

After UACR adjustment (N = 103), the months × PC1 association was substantially attenuated (*P* = 0.792). The months × log UACR interaction was also non-significant (*P* = 0.626). Subgroup analyses (**Supplementary Table 6**) showed a consistent direction of the months × PC1 interaction across all strata examined (age, sex, baseline eGFR, glycemic control, SGLT2i use), with nominal *P*-values (uncorrected for multiplicity). The PC1 × UA three-way interaction was non-significant (*P* = 0.693; **Figure 6**), and the PC2 × SGLT2i interaction showed a trend (*P* = 0.085; **Figure 7**). Stratified LMM (**Supplementary Table 5**) showed nominal SGLT2i effects of *β* = −0.332 (*P* = 0.086) in low PC2, + 0.481 (*P* = 0.069) in medium PC2, and +0.224 (*P* = 0.326) in high PC2 tertiles.

### 3.7 Bridge analysis

**Figure 8:**
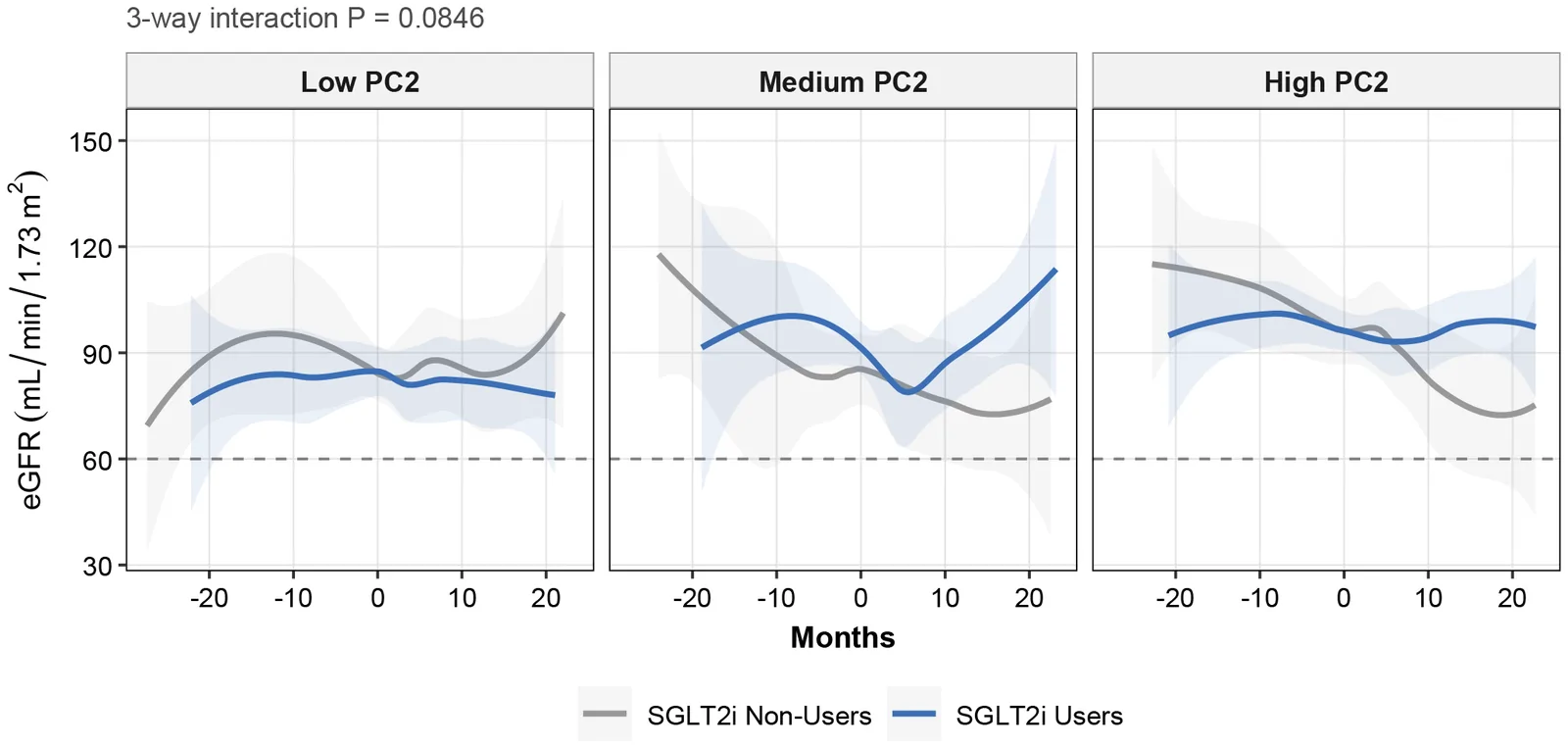
PC scores by WeChat engagement group (N = 145). PC Scores by WeChat Engagement Group (N = 145). G1: consistent follow-up (*n* = 22); G2: new patients (*n* = 74); G3: limited follow-up (*n* = 49). Boxplots show median, interquartile range, and 1.5 × IQR whiskers; overlaid jittered points show individual patient values. PC2 ANOVA *P* = 0.024; after ANCOVA adjustment (age, sex, baseline HbA1c, baseline SBP): PC2 *P* = 0.090, PC1 *P* = 0.005, PC3 *P* = 0.071. The shift in significance after covariate adjustment illustrates the critical importance of confounder control in observational analyses. Given the cross-sectional nature of this analysis, bidirectional confounding between metabolic burden and healthcare engagement cannot be excluded. Abbreviations: ANCOVA, analysis of covariance; ANOVA, analysis of variance; PC, principal component.

PC2 differed across engagement groups in unadjusted ANOVA (*P* = 0.024) but not after ANCOVA adjustment (*P* = 0.090). PC1 became significant post-adjustment (*P* = 0.005; **Table 4**, **Figure 8**), demonstrating that failure to adjust for relevant confounders can both produce spurious associations and obscure genuine ones.

**Table 4:** PC scores by WeChat engagement group.

Group	N	PC1	PC2	PC3
G1 (consistent)	22	–0.06 ± 1.13	0.41 ± 0.89	0.07 ± 0.78
G2 (new)	74	0.03 ± 1.00	0.23 ± 1.13	–0.07 ± 0.99
G3 (limited)	49	0.17 ± 0.97	0.03 ± 1.22	0.34 ± 0.82

Mean ± SD. PC2 ANOVA P=0.024. ANCOVA (adjusted for age, sex, baseline HbA1c, baseline SBP): PC2 P=0.090; PC1 P=0.005; PC3 P=0.071. N = 332 with complete covariate data. Caution: cross-sectional; bidirectional confounding between metabolic burden and healthcare engagement cannot be excluded.

### 3.8 Rapid eGFR decline prediction

**Figure 9:**
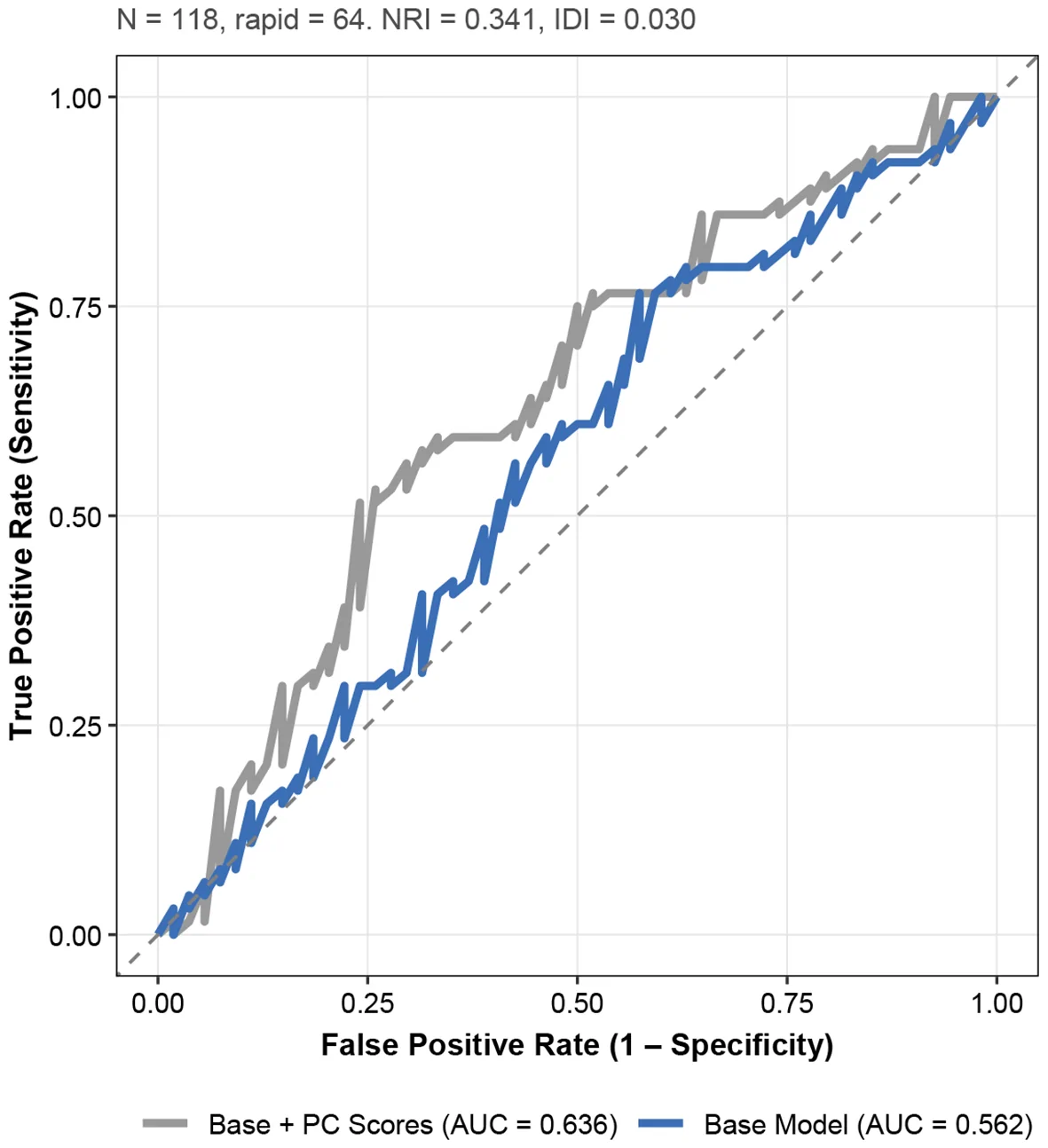
ROC curves for rapid eGFR decline prediction. ROC Curves for Prediction of Rapid eGFR Decline (N = 119, rapid decline = 65, 54.6%). Rapid decline was defined as an annualized eGFR slope *<* −3 mL/min/1.73 m^2^/year. Base model (age + sex + baseline eGFR): AUC = 0.562. Augmented model (+ PC1 + PC2 + PC3): AUC = 0.636 (δAUC = +0.074, LRT *P* = 0.315). Bootstrap-corrected AUC for the augmented model: 0.674 (95% CI 0.588–0.762). The non-significant LRT indicates that the incremental predictive value of PCA dimensions over established predictors (age, sex, baseline eGFR) is modest at best in this cohort. Abbreviations: ROC, receiver operating characteristic; AUC, area under the ROC curve; eGFR, estimated glomerular filtration rate; PC, principal component; LRT, likelihood ratio test; CI, confidence interval.

**Figure 10:**
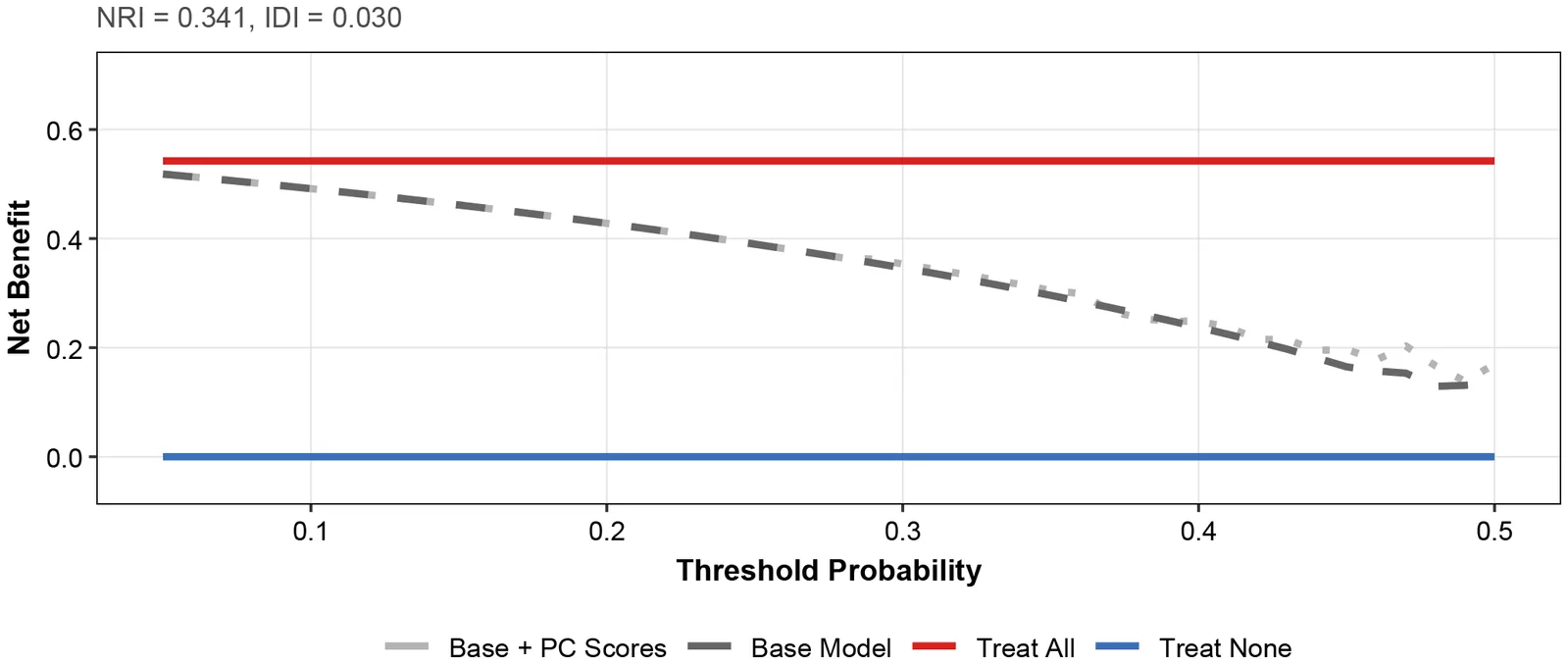
Decision curve analysis. Decision Curve Analysis for Rapid eGFR Decline Prediction. Net benefit is shown across threshold probabilities of 5–50%. The “Treat All” and “Treat None” strategies serve as reference lines. The PCA-augmented model (Base + PC Scores) showed marginally higher net benefit than the base model at clinically relevant threshold probabilities of 5–20%; however, the clinical significance of this improvement is uncertain given the non-significant LRT (*P* = 0.315) and the wide bootstrap confidence interval for AUC (0.588–0.762).

Rapid eGFR decline was observed in 65 of 119 eligible patients (54.6%). The addition of PCA dimensions to the base model yielded a modest, non-statistically significant improvement in discrimination (AUC: 0.562 → 0.636; δAUC = +0.074; LRT *P* = 0.315). Bootstrap-corrected AUC was 0.674 (95% CI 0.588– 0.762; **Supplementary Table 7**; **Figure 9**). Calibration was adequate (HL *P* = 0.771; **Supplementary Figure 2**). Continuous NRI was 0.341, categorical NRI was 0.129, and IDI was 0.030. DCA (**Figure 10**) suggested a marginal net benefit at low threshold probabilities (5–20%), though the clinical significance of this improvement is uncertain given the non-significant LRT. Sensitivity analysis restricted to patients with ≥6 months of follow-up (N = 74, 38 events) yielded consistent results (AUC: 0.571 → 0.662).

In the UACR-available subcohort (N = 35, 18 events; **Supplementary Table 8**), the base model with log UACR achieved AUC of 0.837, increasing to 0.925 with PCA dimensions (bootstrap-corrected: 0.959, 95% CI 0.866–1.000). Given N = 35 with 18 events, this model suffers from quasi-complete separation; the AUC of 0.925 is almost certainly overestimated and should not be cited as evidence of predictive utility. This subcohort analysis is presented solely for completeness and should not inform any clinical or research conclusion.

### 3.9 CKD progression

Only 6 CKD progression events were observed among 100 at-risk patients (6.0%). Cox regression was not performed due to insufficient events (19).

## 4 Discussion

In this PCA-based analysis of 621 Chinese patients with T2D, three interpretable metabolic phenotype dimensions emerged: a Blood Pressure Axis (PC1), a Metabolic Syndrome Axis (PC2), and a Renal Function Axis (PC3). PC1 was associated with differential eGFR trajectories, and this association was substantially attenuated after UACR adjustment—a finding that generates the hypothesis that blood pressureassociated renal function decline may involve albuminuric pathways, though this cannot be confirmed in an observational design. Temporal validation confirmed the stability of PCA loading structures across time periods, while predictive model generalization was limited, consistent with the modest, non-significant improvement observed in the primary analysis.

### 4.1 PCA as a phenotyping framework

A key methodological contribution of this study is the rigorous validation of the PCA-based phenotyping framework. The Hopkins statistic (0.994) provided strong evidence against a clustered data structure, empirically justifying the choice of a dimensional approach over discrete clustering. Temporal validation demonstrated that the PCA loading structures were highly stable across pre- and post-intervention cohorts (PC1 *r* = 0.961, PC2 *r* = 0.928, PC3 *r* = −0.976), which supports the temporal robustness of the identified metabolic dimensions. These findings are important because they suggest that the three PCA dimensions capture stable, underlying physiological processes rather than statistical artifacts of a particular sample.

The comparison of 3-component and 4-component models revealed a tension between statistical performance and interpretability. The 4-component model yielded a statistically significant improvement in predictive discrimination (LRT *P* = 0.001), which we report transparently. However, PC4 was dominated by HbA1c and LDL-C—variables already partially represented in PC2—and introduced cross-loadings without a distinct additional physiological interpretation. Retaining a component solely on statistical grounds, when its biological meaning is unclear, risks overfitting and reduces clinical translatability. We therefore retained the 3-component solution, consistent with the principle that PCA dimensions should correspond to interpretable pathophysiological domains (5). We acknowledge that this judgment involves an element of subjectivity and that the 4-component solution may be preferable in other populations or for different clinical endpoints.

### 4.2 Physiological significance of PCA dimensions

The three-component structure is biologically coherent and aligns with established pathophysiological frameworks. PC1, dominated by SBP and DBP, captures the hemodynamic dimension of cardiometabolic disease. The strong, specific association with hypertension (OR ≈ 30) confirms construct validity. The observed association of PC1 with both eGFR decline and macroalbuminuria is biologically plausible: elevated systemic pressure promotes glomerular hyperfiltration and podocyte stress, which may contribute to both albumin leakage and GFR loss through glomerulosclerosis and tubulointerstitial fibrosis (20, 21). However, the cross-sectional nature of UACR assessment and the observational design preclude any inference about the temporal sequence of these events.

PC2 reflects the insulin resistance/dyslipidemia cluster characterized by low HDL-C, elevated TG, and moderate contributions from HbA1c and UA. This axis captures the “metabolic syndrome” dimension first described by Reaven (22). The borderline association with moderate albuminuria (OR 1.43, *P* = 0.052) but not with eGFR decline in the short term suggests that this metabolic dimension may require longer exposure to manifest renal consequences, or that the high SGLT2i use in higher PC2 tertiles may have attenuated metabolic syndrome-related renal effects.

PC3 is inevitably dominated by eGFR and UA, reflecting baseline renal function. Its inverse associations with CKD, hyperuricemia, and albuminuria are largely tautological, confirming that this axis serves primarily as a continuous renal function marker rather than an independent phenotypic dimension.

### 4.3 Biological mechanisms linking metabolic dimensions to renal outcomes

Several molecular pathways may link the identified metabolic dimensions to renal function decline. mTORC1, a central nutrient-sensing kinase, integrates amino acid and growth factor signals to regulate cellular metabolism, as demonstrated by studies showing that amino acid–Rab1A–mTORC1–PDX1 signaling controls *β*-cell function and glucose homeostasis (23, 24). These nutrient-sensing pathways—including mTOR, AMP-activated protein kinase (AMPK), and NAD^+^-dependent sirtuins (SIRT1)—converge on transcriptional programs that enhance autophagy, proteostasis, mitochondrial function, and stress resistance, as comprehensively reviewed in the context of dietary restriction (25). In the kidney, these same pathways have been independently implicated in diabetic kidney disease (DKD): mTOR hyperactivation contributes to podocyte injury through impaired autophagy and mesangial expansion, while AMPK and SIRT1 activation restores autophagic flux and attenuates renal fibrosis in experimental models. We emphasize that the foundational mechanistic studies cited above are primarily cell- and animal-based; our contribution lies in the clinical application of metabolic phenotyping in a real-world T2D cohort, and the molecular pathways discussed here represent hypotheses requiring dedicated experimental validation in diabetes-specific renal models. SGLT2 inhibitors may partially interrupt these pathways by restoring tubuloglomerular feedback, reducing proximal tubular metabolic stress, and activating nutrient-deprivation signaling cascades including AMPK and SIRT1, which may contribute to their renoprotective effects. These relationships are summarized in **Supplementary Figure 5**.

### 4.4 Clinical implications and effect sizes

The annualized effect of PC1 on eGFR decline (−1.62 mL/min/1.73 m^2^/year per 1-SD; approximately −3.55 mL/min/1.73 m^2^/year between extreme tertiles) is comparable in magnitude to the renoprotective effect of intensive blood pressure control reported in landmark trials (20, 26). At the individual level, this difference is unlikely to be clinically discernible over the short follow-up period (13 months), but if sustained over 5–10 years, it would translate into a clinically meaningful gap in renal function. This effect size underscores the importance of blood pressure control as a modifiable determinant of renal outcomes in T2D, independent of glycemic status.

The OR for macroalbuminuria (1.76 per SD of PC1) represents a clinically meaningful effect, indicating that patients 1 SD above versus below the mean on the blood pressure axis have a nearly 2-fold higher odds of severe albuminuria. This effect is comparable to established albuminuria risk factors and supports the potential utility of PC1 as a continuous risk marker.

### 4.5 Comparison with existing renal risk prediction models

The predictive performance of the PCA-augmented model (AUC 0.636, bootstrap-corrected 0.674) is modest compared with established DKD risk models such as the Kidney Failure Risk Equation (KFRE) and the ADVANCE risk score, which achieve AUC values of 0.85–0.92 in their respective derivation cohorts. A direct comparison is limited by differences in study populations, follow-up duration, and predictor availability (particularly UACR). The limited incremental value of PCA dimensions in our study (LRT *P* = 0.315) may reflect two factors: (1) the short follow-up duration, which limits the ability to distinguish pathological decline from eGFR variability, and (2) the fact that baseline eGFR and age— already included in the base model—capture a substantial portion of renal risk. Whether PCA dimensions improve prediction in cohorts with longer follow-up and complete UACR data remains an open question for future investigation.

### 4.6 Limitations

Several limitations warrant emphasis and contextualize the generalizability of the findings.

First, and most critically, this is a single-center study from a regional hospital in Sichuan Province, China, without independent external validation. While we performed extensive internal validation (bootstrap resampling, temporal validation, subgroup analyses, calibration assessment, sensitivity analyses), the generalizability of our PCA loading structure and prediction model to other populations—particularly those with different demographic profiles, comorbidity patterns, or treatment practices—remains unknown. Notably, temporal validation within our own institution demonstrated that while PCA loading structures were highly stable across time periods (all *r >* 0.92), the predictive model for rapid eGFR decline failed to generalize (AUC dropped from 0.635 in training to 0.543 in testing; **Supplementary Figure 3**). This disconnect between structural stability and predictive failure is a critical finding: it demonstrates that even internally consistent phenotypic dimensions may not confer robust predictive utility, and it strongly cautions against any clinical application of the prediction model without independent external validation in larger, multi-center cohorts. We have evaluated several candidate external datasets, including the China Health and Retirement Longitudinal Study (CHARLS; lacks UACR data), the National Health and Nutrition Examination Survey (NHANES; United States population with uncertain cross-population generalizability), and the China Cardiometabolic Disease and Cancer Cohort (4C Study; provides longitudinal metabolic and urine biomarker data in a Chinese population). Formal external validation in the 4C Study or an equivalent multi-center cohort is the immediate next phase of this research program.

Second, the short follow-up duration (∼13 months post-intervention) limits the ability to distinguish pathological eGFR decline from acute fluctuations due to dehydration, infection, medication changes, or laboratory variation. The high proportion of patients classified as rapid decliners (54.6%) suggests that the −3 mL/min/1.73 m^2^/year threshold, conventionally applied to longer-term studies (18), may be overly sensitive in a short observation window.

Third, UACR data were available in only 17.9% of observations (5.9% at baseline), substantially limiting the UACR-adjusted analyses and precluding robust KDIGO-based risk stratification. The UACR prediction subcohort (N = 35, 18 events) yielded unstable estimates due to quasi-complete separation (bootstrap-corrected AUC 0.959 with a CI spanning 0.866–1.000). We reiterate that these results are statistically unstable, almost certainly overestimated, and included solely for transparent reporting; they should not be cited as evidence of predictive utility or used to inform any clinical conclusion. The absence of complete UACR data—a cornerstone of DKD risk assessment (8)—is a major limitation of this study.

Fourth, we did not systematically account for medications beyond SGLT2i that may influence renal outcomes, including metformin (used by the majority of patients), insulin, RAAS inhibitors, and statins. The absence of detailed medication dosing, duration, and adherence data precludes rigorous pharmacoepidemiological adjustment. The observed associations between PCA dimensions and renal outcomes may be confounded by differential prescribing patterns across phenotype strata.

Fifth, CKD progression events were insufficient (*<*10) for meaningful survival analysis, precluding time-to-event analyses that are standard in the DKD literature. Sixth, the longitudinal eGFR cohort (N = 162) is relatively small, limiting statistical power for interaction analyses and increasing the risk of type II error. Seventh, the PCA-eligible cohort differed systematically from excluded patients (higher HbA1c, SBP, DBP, and longer diabetes duration), introducing potential selection bias toward patients with more complete clinical data and possibly greater disease severity. Eighth, the absence of insulin and C-peptide data precludes direct comparison with international clustering studies (3). Ninth, the WeChat engagement bridge analysis was cross-sectional and cannot distinguish whether adverse metabolic profiles lead to lower healthcare engagement or whether lower engagement exacerbates metabolic derangements; a prospective, intervention-based study is needed to disentangle the causal direction. Tenth, the study population is exclusively Chinese, and the generalizability of PCA-derived metabolic dimensions to other ethnic groups requires investigation. Eleventh, the retrospective, observational design cannot establish causal or mediating relationships; all reported associations should be considered hypothesis-generating.

### 4.7 Strengths

Despite these limitations, the study has notable strengths: (1) comprehensive methodological validation of the PCA approach, including parallel analysis, Promax rotation sensitivity, 3-vs-4-component comparison, Hopkins statistic, LPA, MICE-derived ICC, complete-case sensitivity, and temporal validation; (2) the integration of UACR data, which enabled KDIGO-based risk stratification and the hypothesis-generating observation of UACR-associated attenuation of the PC1–eGFR relationship; (3) the translation of statistical coefficients into clinically interpretable annualized effect sizes; (4) the use of appropriately specified LMM to model within-patient correlation; (5) bootstrap internal validation with calibration assessment; (6) subgroup analyses demonstrating the consistency of key findings across clinically relevant strata; (7) the transparent reporting of both PCA loading stability and predictive model generalization failure in temporal validation; and (8) the application of PCA to routine, widely available clinical variables, enhancing translational potential.

## Data Availability

The datasets analyzed during the current study are not publicly available due to patient privacy protection. De‑identified data may be made available from the corresponding author upon reasonable request and with appropriate institutional approvals. Complete PCA loading matrices and model specifications are provided in the **Supplementary Material** to facilitate independent replication.

## Supplementary material

Supplementary Table 1 Four-Component PCA Loadings (per Parallel Analysis) PC4 explains 12.6% of total variance. Dominant loadings: HbA1c (%) and LDL-C (mmol/L). No clear physiological interpretation beyond PC2; 3-component solution retained on grounds of parsimony and interpretability.

Supplementary Table 2 Comparison of 3-Component vs. 4-Component PCA ModelsThe 4-component model yielded a statistically significant improvement in predictive discrimination; however, PC4 was dominated by variables already represented in PC2, introduced cross-loadings, and lacked a distinct physiological interpretation. The 3-component solution was retained for parsimony. LRT: likelihood ratio test.

Supplementary Table 3 Stratified SGLT2i × months Effects by PC2 TertileStratified LMM: eGFR ~ months **×** SGLT2i + age + sex + HbA1c + (1 | patient_id). Nominal *P*-values (uncorrected for multiplicity). These analyses are exploratory and underpowered.

Supplementary Table 4 Winsorization Before/After ComparisonWinsorization at 1st and 99th percentiles. Out = number of outliers beyond thresholds. Means were minimally affected (all changes < 0.05 SD).

Supplementary Table 5 Prediction Model Performance Metrics Bootstrap: 1,000 resamples. Categorical NRI cutoffs: <30%, 30–60%, >60% predicted risk. Sensitivity AUC excludes patients with <6 months follow-up (N = 74, 38 events). LRT: likelihood ratio test; NRI: net reclassification improvement; IDI: integrated discrimination improvement; HL: Hosmer-Lemeshow test.

Supplementary Table 6 Baseline UACR and KDIGO Characteristics UACR available in subset of patients with simultaneous urine creatinine and microalbumin measurements. KDIGO 2024 categories: A1 = normal to mildly increased; A2 = moderately increased; A3 = severely increased. IQR: interquartile range.

Supplementary Table 7 Prediction Model Performance (UACR Subcohort) Bootstrap: 1,000 resamples. **Caution:** Extremely small sample (N = 35, 18 events). AUC almost certainly overestimated due to quasi-complete separation. Included solely for transparent reporting; should not be cited as evidence of predictive utility

Supplementary Table 8 Comparison of Included vs. Excluded Participants Mean ± SD. *P* values from independent-samples *t*-tests. PCA inclusion required ≥6 of 9 metabolic variables. Excluded patients had lower HbA1c, SBP, DBP, and shorter diabetes duration, suggesting that the PCA cohort was biased toward patients with more complete clinical data and possibly greater metabolic disease severity.

Supplementary Table 9 Stratified months **×** PC1 Effects Across Subgroups LMM stratified by subgroup: eGFR ~ months **×** PC1 + PC2 + PC3 + age + sex + HbA1c + med_sglt2i + (1 | patient_id). Nominal *P*-values; not adjusted for multiplicity. The Sex stratum (Male vs. Female) is omitted because sex is collinear within sex-specific strata; the overall model includes sex as a covariate. The consistent direction of the months **×** PC1 interaction across all examined strata supports the robustness of the primary finding. Subgroups with <15 patientswere excluded.

Supplementary Figure 1 LPA Model Selection **Supplementary Figure 1**. Latent Profile Analysis (LPA) Model Selection. Bayesian Information Criterion (BIC) for Gaussian mixture models with 1–6 clusters. The optimal solution was *G* = 4 clusters (lowest BIC). Hopkins statistic = 0.994, strongly favoring a continuous (dimensional) data structure over discrete clustering. This contrast between LPA (which identifies clusters by statistical optimization regardless of data structure) and the Hopkins statistic (which directly tests for clustering tendency) provides empirical support for the PCA-based dimensional approach adopted in this study. Abbreviations: BIC, Bayesian Information Criterion; LPA, latent profile analysis; PCA, principal component analysis.

Supplementary Figure 2 KDIGO Risk Stratification by PC1 Tertile. **Supplementary Figure 2**. KDIGO 2024 Risk Stratification by PC1 Tertile (N = 190 with complete eGFR and UACR data). Stacked bar chart showing the proportion of patients in each KDIGO risk category (Low Risk, Moderately Increased Risk, High Risk, Very High Risk) across PC1 tertiles. Higher PC1 tertile (greater blood pressure burden) was associated with a shift toward greater KDIGO risk, consistent with the known role of hypertension as a risk factor for CKD progression. However, these findings are limited by the small proportion of patients with complete KDIGO data (190/621, 30.6%). Abbreviations: KDIGO, Kidney Disease: Improving Global Outcomes; eGFR, estimated glomerular filtration rate; UACR, urinary albumin-to-creatinine ratio; PC1, principal component 1 (Blood Pressure Axis).

Supplementary Figure 3 Calibration Plot. **Supplementary Figure 3**. Calibration Plot for the PCA-Augmented Rapid eGFR Decline Prediction Model. Predicted probabilities are plotted against observed proportions across quintiles of predicted risk. The dashed diagonal line represents perfect calibration. Point size is proportional to the number of patients per bin. The Loess smoothing line (blue) with 95% confidence band indicates acceptable calibration across the range of predicted probabilities. Hosmer-Lemeshow goodness-of-fit test: *P* = 0.771, indicating no significant miscalibration. Abbreviations: PCA, principal component analysis; eGFR, estimated glomerular filtration rate. Metabolic Phenotype Dimensions & Renal Outcomes in T2D

Supplementary Figure 4 Integrative Conceptual Framework. Schematic illustration linking PCA-derived metabolic phenotype dimensions (top row) to their respective clinical outcomes and correlates (middle row), and to potential underlying molecular mechanisms (bottom panel). PC1 (Blood Pressure Axis) is associated with accelerated eGFR decline, macroalbuminuria (OR 1.76), and KDIGO risk progression. PC2 (Metabolic Syndrome Axis) is associated with dyslipidemia (OR 3.44), hyperuricemia (OR 3.18), and poor glycemic control. PC3 (Renal Function Axis) reflects baseline renal function and is inversely associated with albuminuria and CKD. Molecular pathways potentially linking these dimensions to renal outcomes include RAAS activation, endothelial dysfunction, oxidative stress, inflammation, podocyte injury, mesangial expansion, tubulointerstitial fibrosis, mTOR signaling, AMPK/SIRT1, autophagy, and mitochondrial dysfunction. SGLT2 inhibitors may partially interrupt these pathways via tubuloglomerular feedback restoration and nutrient-deprivation signaling cascades. Abbreviations: RAAS, renin-angiotensinaldosterone system; mTOR, mechanistic target of rapamycin; AMPK, AMP-activated protein kinase; SIRT1, sirtuin 1; OR, odds ratio.

Supplementary Figure 5 Temporal Validation (Pre vs Post). Temporal Validation of Rapid eGFR Decline Prediction Model. ROC curves for the prediction of rapid eGFR decline in an independent post-intervention test cohort (N = 42, 22 events) using a model trained exclusively on pre-intervention data (N = 76, 42 events). PCA loading structures demonstrated excellent temporal stability (PC1 *r* = 0.961, PC2 *r* = 0.928, PC3 *r* = −0.976, where the sign reversal for PC3 reflects the known sign ambiguity of PCA). However, the incremental predictive value of PCA scores did not generalize (training AUC: 0.551 → 0.635; testing AUC: 0.575 → 0.543; generalization gap for +PC model: −0.092), consistent with the modest, non-significant improvement observed in the primary analysis and reinforcing the need for independent external validation. Abbreviations: ROC, receiver operating characteristic; AUC, area under the ROC curve; eGFR, estimated glomerular filtration rate; PC, principal component.
