# Supplementary material for: Metabolic phenotype dimensions and longitudinal renal function in type 2 diabetes: a principal component analysis-based approach with UACR integration
Source: Front Endocrinol (Lausanne). 2026 Aug 31;17:1929060. doi: 10.3389/fendo.2026.1929060 (PMC13572980; doi:10.3389/fendo.2026.1929060)
Supplement: Supplementary file 1 [file SupplementaryFile1.zip › Supplementary_Table_S1.pdf]

Supplementary Table S1: Comparison of Included vs Excluded Participants

Variable	Included	Excluded	P
age	57.2 +/- 10.3 (N = 619)	57.6 +/- 9.7 (N = 806)	0.475
hba1c	9.1 +/- 2.6 (N = 580)	8.3 +/- 2.2 (N = 481)	<0.001
bmi	25.2 +/- 3.5 (N = 341)	25.2 +/- 3.9 (N = 94)	0.947
sbp_clean	132.2 +/- 19.7 (N = 375)	125.5 +/- 14.5 (N = 177)	<0.001
dbp_clean	85.0 +/- 11.0 (N = 374)	80.1 +/- 8.9 (N = 177)	<0.001
egfr	92.9 +/- 19.7 (N = 596)	93.4 +/- 19.9 (N = 136)	0.822
uric_acid_clean	326.0 +/- 96.4 (N = 596)	323.4 +/- 95.1 (N = 138)	0.781
dm_duration_months	61.9 +/- 62.6 (N = 262)	41.2 +/- 52.1 (N = 134)	<0.001
