# Supplementary material for: Metabolic phenotype dimensions and longitudinal renal function in type 2 diabetes: a principal component analysis-based approach with UACR integration
Source: Front Endocrinol (Lausanne). 2026 Aug 31;17:1929060. doi: 10.3389/fendo.2026.1929060 (PMC13572980; doi:10.3389/fendo.2026.1929060)
Supplement: Supplementary file 1 [file SupplementaryFile1.zip › Supplementary_Table_S2.pdf]

# Supplementary Table S2: Winsorization Before/After Comparison

| Variable | N | N_Outliers_Low | N_Outliers_High | Before_Min | Before_Max | After_Min | After_Max |
|---|---|---|---|---|---|---|---|
| HbA1c (%) | 580 | 3 | 6 | 4.50 | 17.00 | 4.90 | 15.32 |
| BMI (kg/m^2) | 341 | 4 | 4 | 16.80 | 36.76 | 18.69 | 34.82 |
| SBP (mmHg) | 375 | 4 | 4 | 70.00 | 216.00 | 94.74 | 183.26 |
| DBP (mmHg) | 374 | 3 | 3 | 55.00 | 128.00 | 62.00 | 116.00 |
| LDL-C (mmol/L) | 597 | 6 | 6 | 0.28 | 9.73 | 0.50 | 5.51 |
| HDL-C (mmol/L) | 597 | 6 | 6 | 0.23 | 2.76 | 0.77 | 2.35 |
| TG (mmol/L) | 597 | 6 | 6 | 0.24 | 24.49 | 0.43 | 18.50 |
| eGFR (mL/min/1.73m^2) | 596 | 6 | 6 | 3.10 | 147.21 | 37.26 | 124.86 |
| UA (umol/L) | 596 | 6 | 6 | 115.00 | 759.50 | 145.55 | 610.50 |
| log UACR | 184 | 2 | 2 | -0.54 | 8.41 | -0.16 | 7.93 |
