# Supplementary material for: Metabolic phenotype dimensions and longitudinal renal function in type 2 diabetes: a principal component analysis-based approach with UACR integration
Source: Front Endocrinol (Lausanne). 2026 Aug 31;17:1929060. doi: 10.3389/fendo.2026.1929060 (PMC13572980; doi:10.3389/fendo.2026.1929060)
Supplement: Supplementary file 1 [file SupplementaryFile1.zip › Supplementary_Table_S3.pdf]

Supplementary Table S3: Four-Component PCA Loadings

Variable	PC1	PC2	PC3	PC4
HbA1c (%)	0.076	0.338	-0.213	0.768
BMI (kg/m^2)	0.156	0.165	0.436	-0.391
SBP (mmHg)	0.907	-0.092	0.048	-0.110
DBP (mmHg)	0.887	0.096	0.071	0.105
LDL-C (mmol/L)	-0.015	-0.402	0.246	0.652
HDL-C (mmol/L)	0.165	-0.704	-0.270	-0.065
TG (mmol/L)	0.132	0.761	-0.085	-0.065
eGFR (mL/min/1.73m^2)	-0.071	0.207	-0.709	-0.023
UA (umol/L)	-0.009	0.270	0.789	-0.035
