# Supplementary material for: Metabolic phenotype dimensions and longitudinal renal function in type 2 diabetes: a principal component analysis-based approach with UACR integration
Source: Front Endocrinol (Lausanne). 2026 Aug 31;17:1929060. doi: 10.3389/fendo.2026.1929060 (PMC13572980; doi:10.3389/fendo.2026.1929060)
Supplement: Supplementary file 1 [file SupplementaryFile1.zip › Supplementary_Table_S4.pdf]

Supplementary Table S4: Comparison of 3- vs 4-Component PCA Models

Metric	3-Component	4-Component
Variance explained (%)	53.1	65.7
AUC (3-PC model)	0.562	—
AUC (4-PC model)	—	0.699
LRT P (3-PC vs 4-PC)	—	0.0011
PC4 dominant loadings (|r| > 0.3)	—	HbA1c (%), BMI (kg/m^2), LDL-C (mmol/L)
Cross-loadings (>1 PC with |r| > 0.3)	None	HbA1c (%), BMI (kg/m^2), LDL-C (mmol/L)
