# Supplementary material for: Metabolic phenotype dimensions and longitudinal renal function in type 2 diabetes: a principal component analysis-based approach with UACR integration
Source: Front Endocrinol (Lausanne). 2026 Aug 31;17:1929060. doi: 10.3389/fendo.2026.1929060 (PMC13572980; doi:10.3389/fendo.2026.1929060)
Supplement: Supplementary file 1 [file SupplementaryFile1.zip › Supplementary_Table_S5.pdf]

Supplementary Table S5: Stratified SGLT2i Effects by PC2 Tertile

PC2 Tertile	N	months x SGLT2i (beta)	SE	P
Low PC2	55	-0.3316	0.1903	0.086
Medium PC2	55	0.4807	0.2601	0.069
High PC2	52	0.2242	0.2271	0.326
