# Supplementary material for: Metabolic phenotype dimensions and longitudinal renal function in type 2 diabetes: a principal component analysis-based approach with UACR integration
Source: Front Endocrinol (Lausanne). 2026 Aug 31;17:1929060. doi: 10.3389/fendo.2026.1929060 (PMC13572980; doi:10.3389/fendo.2026.1929060)
Supplement: Supplementary file 1 [file SupplementaryFile1.zip › Supplementary_Table_S6.pdf]

Supplementary Table S6: Stratified months x PC1 Effects Across Subgroups

Stratum	Level	N_patients	N_obs	months x PC1 (beta)	SE	P
Age	Older	84	208	-0.1740	0.1078	0.108
Age	Younger	78	207	-0.1301	0.0716	0.071
Baseline eGFR	eGFR >= 60	151	364	-0.0970	0.0499	0.053
Baseline eGFR	eGFR < 60	28	51	-0.0980	0.3648	0.790
Glycemic Control	HbA1c >= 8%	99	151	-0.0556	0.1072	0.605
Glycemic Control	HbA1c < 8%	118	209	-0.1520	0.0785	0.054
SGLT2i Use	SGLT2i Users	109	215	-0.0871	0.0870	0.319
SGLT2i Use	SGLT2i Non-Users	103	200	-0.1497	0.0863	0.085
