# Supplementary material for: Metabolic phenotype dimensions and longitudinal renal function in type 2 diabetes: a principal component analysis-based approach with UACR integration
Source: Front Endocrinol (Lausanne). 2026 Aug 31;17:1929060. doi: 10.3389/fendo.2026.1929060 (PMC13572980; doi:10.3389/fendo.2026.1929060)
Supplement: Supplementary file 1 [file SupplementaryFile1.zip › Supplementary_Table_S7.pdf]

Supplementary Table S7: Prediction Model Performance Metrics

Metric	Value
AUC (Base)	0.562
AUC (+PC)	0.636
Delta AUC	0.073
Bootstrap AUC (+PC)	0.674
Bootstrap 95% CI	0.588–0.762
Continuous NRI	0.341
Categorical NRI	0.129
IDI	0.0303
LRT P	0.3146
HL P	0.7708
Sensitivity AUC Base (>=6mo)	0.571
Sensitivity AUC +PC (>=6mo)	0.662
