# Supplementary material for: Metabolic phenotype dimensions and longitudinal renal function in type 2 diabetes: a principal component analysis-based approach with UACR integration
Source: Front Endocrinol (Lausanne). 2026 Aug 31;17:1929060. doi: 10.3389/fendo.2026.1929060 (PMC13572980; doi:10.3389/fendo.2026.1929060)
Supplement: Supplementary file 1 [file SupplementaryFile1.zip › Supplementary_Table_S8.pdf]

Supplementary Table S8: Prediction Performance (UACR Subcohort)

Metric	Value
AUC (Base+UACR)	0.837
AUC (+PC+UACR)	0.925
Delta AUC	0.088
Bootstrap AUC	0.959
Bootstrap 95% CI	0.866–1.000
N	35
Events	18
