# Supplementary material for: Metabolic phenotype dimensions and longitudinal renal function in type 2 diabetes: a principal component analysis-based approach with UACR integration
Source: Front Endocrinol (Lausanne). 2026 Aug 31;17:1929060. doi: 10.3389/fendo.2026.1929060 (PMC13572980; doi:10.3389/fendo.2026.1929060)
Supplement: Supplementary file 1 [file SupplementaryFile1.zip › Supplementary_Table_S9.pdf]

Supplementary Table S9: Baseline UACR and KDIGO Characteristics

Metric	Value
N (total)	621
N (UACR available)	184
Median UACR (mg/g)	22
UACR IQR	8–102
A1 (<30 mg/g) %	57.6%
A2 (30-300 mg/g) %	27.7%
A3 (>300 mg/g) %	14.7%
