# Supplementary material for: Metabolic phenotype dimensions and longitudinal renal function in type 2 diabetes: a principal component analysis-based approach with UACR integration
Source: Front Endocrinol (Lausanne). 2026 Aug 31;17:1929060. doi: 10.3389/fendo.2026.1929060 (PMC13572980; doi:10.3389/fendo.2026.1929060)

Supplementary Figure S4. KDIGO Risk Stratification by PC1 Tertile

N = 190 with complete eGFR + UACR

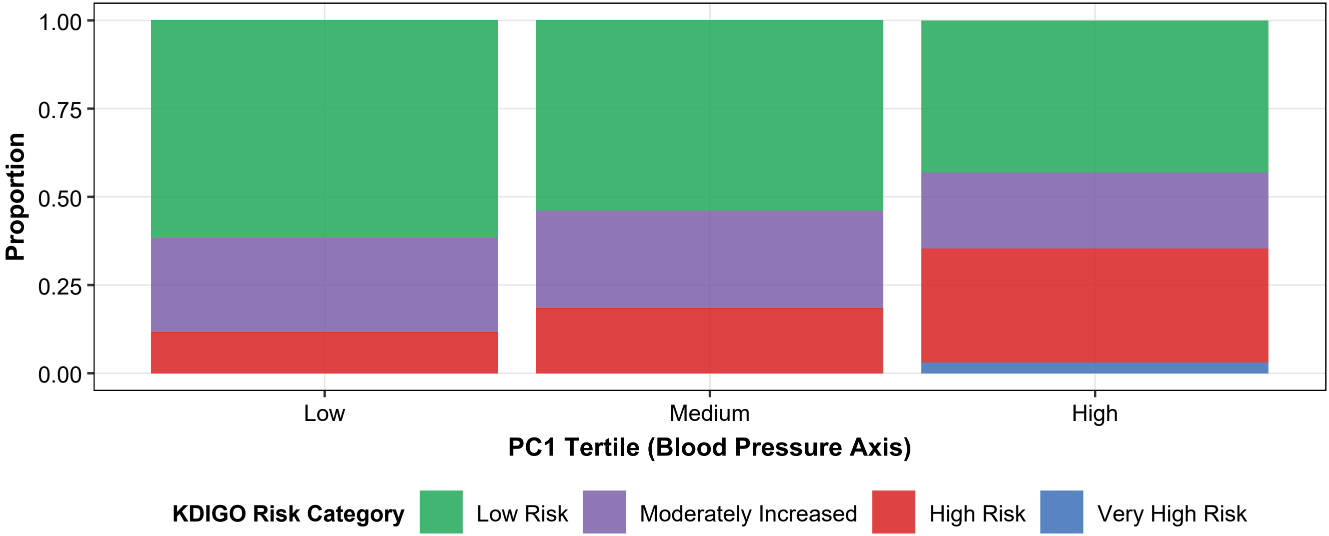

Supplement: Supplementary file 2 [file SupplementaryFile2.zip › Supplementary_Figure_S4.pdf]
