# Supplementary material for: Metabolic phenotype dimensions and longitudinal renal function in type 2 diabetes: a principal component analysis-based approach with UACR integration
Source: Front Endocrinol (Lausanne). 2026 Aug 31;17:1929060. doi: 10.3389/fendo.2026.1929060 (PMC13572980; doi:10.3389/fendo.2026.1929060)
Supplement: Supplementary file 2 [file SupplementaryFile2.zip › Supplementary_Figure_S5.pdf]

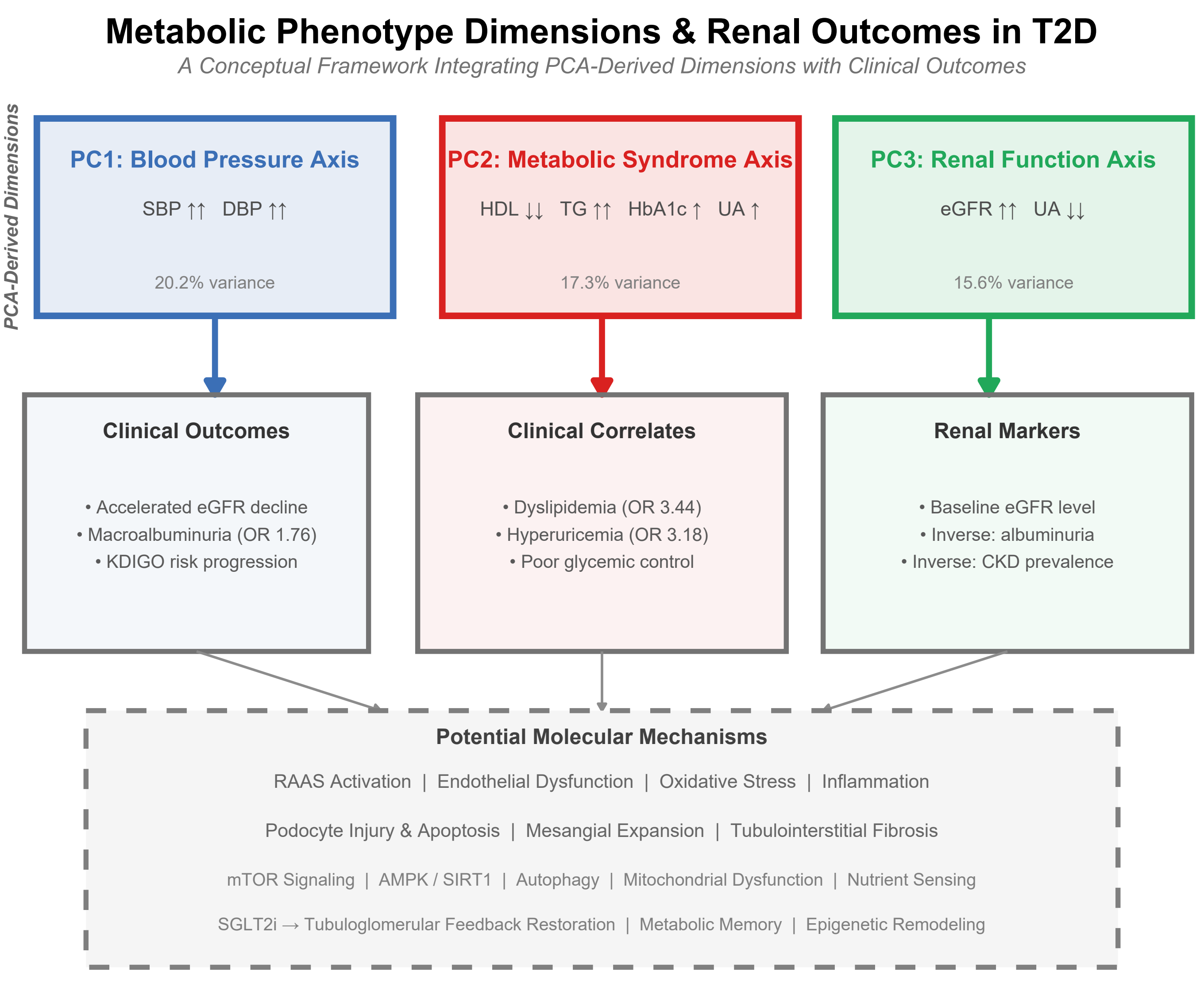
Metabolic Phenotype Dimensions & Renal Outcomes in T2D
A Conceptual Framework Integrating PCA-Derived Dimensions with Clinical Outcomes
PCA-Derived Dimensions
PC1: Blood Pressure Axis
SBP ↑↑ DBP ↑↑
20.2% variance
PC2: Metabolic Syndrome Axis
HDL ↓↓ TG ↑↑ HbA1c ↑ UA ↑
17.3% variance
PC3: Renal Function Axis
eGFR ↑↑ UA ↓↓
15.6% variance
Clinical Outcomes
• Accelerated eGFR decline
• Macroalbuminuria (OR 1.76)
• KDIGO risk progression
Clinical Correlates
• Dyslipidemia (OR 3.44)
• Hyperuricemia (OR 3.18)
• Poor glycemic control
Renal Markers
• Baseline eGFR level
• Inverse: albuminuria
• Inverse: CKD prevalence
Potential Molecular Mechanisms
RAAS Activation | Endothelial Dysfunction | Oxidative Stress | Inflammation
Podocyte Injury & Apoptosis | Mesangial Expansion | Tubulointerstitial Fibrosis
mTOR Signaling | AMPK / SIRT1 | Autophagy | Mitochondrial Dysfunction | Nutrient Sensing
SGLT2i → Tubuloglomerular Feedback Restoration | Metabolic Memory | Epigenetic Remodeling
